# Genetic Diversity of Non-O157 Shiga Toxin-Producing *Escherichia coli* Recovered From Patients in Michigan and Connecticut

**DOI:** 10.3389/fmicb.2020.00529

**Published:** 2020-03-31

**Authors:** Heather M. Blankenship, Rebekah E. Mosci, Quyen Phan, John Fontana, James. T. Rudrik, Shannon D. Manning

**Affiliations:** ^1^Department of Microbiology and Molecular Genetics, Michigan State University, East Lansing, MI, United States; ^2^Connecticut Department of Public Health, Hartford, CT, United States; ^3^Bureau of Laboratories, Michigan Department of Health and Human Services, Lansing, MI, United States

**Keywords:** Shiga toxin, *Escherichia coli*, epidemiology, multilocus sequence typing, clustered regularly interspaced repeats, genotyping

## Abstract

Shiga toxin-producing *Escherichia coli* (STEC) are important foodborne pathogens and non-O157 serotypes have been gradually increasing in frequency. The non-O157 STEC population is diverse and is often characterized using serotyping and/or multilocus sequence typing (MLST). Although spacers within clustered regularly interspaced repeat (CRISPR) regions were shown to comprise horizontally acquired DNA elements, this region does not actively acquire spacers in STEC. Hence, it is useful for further characterizing non-O157 STEC and examining relationships between strains. Our study goal was to evaluate the genetic relatedness of 41 clinical non-O157 isolates identified in Michigan between 2001 and 2005 while comparing to 114 isolates from Connecticut during an overlapping time period. Whole genome sequencing (WGS) was performed, and sequences were extracted for serotyping, MLST and CRISPR analysis. Phylogenetic analysis of MLST and CRISPR data was performed using the Neighbor joining and unweighted pair group method with arithmetic mean (UPGMA) algorithms, respectively. In all, 29 serogroups were identified; eight were unique to Michigan and 13 to Connecticut. “Big-six” serogroup frequencies were similar by state (Michigan: 73.2%, Connecticut: 81.6%), though STEC O121 was not found in Michigan. The distribution of sequence types (STs) and CRISPR profiles was also similar across states. Interestingly, big-six serogroups such as O103 and O26, grouped into different STs located on distinct branches of the phylogeny, further confirming that serotyping alone is not adequate for evaluating strain relatedness. Comparatively, the CRISPR analysis identified 361 unique spacers that grouped into 80 different CRISPR profiles. CRISPR spacers 231 and 317 were isolated from 79.2% (*n* = 118) and 59.1% (*n* = 88) of strains, respectively, regardless of serogroup and ST. Spacer profiles clustered according to the MLST analysis, though some discrepancies were noted. Indeed, use of both MLST and CRISPR typing enhanced the discriminatory power when compared to the use of each tool separately. These data highlight the genetic diversity of clinical STEC from different locations and show that CRISPR profiling can be used alongside MLST to discriminate related strains. Use of targeted sequencing approaches are particularly helpful for sites without WGS capabilities and can help define which strains require additional characterization using more discriminatory methods.

## Introduction

Shiga toxin-producing *Escherichia coli* (STEC) is a leading foodborne pathogen in the United States that was estimated to cause 265,000 illnesses and more than 3,600 hospitalizations each year (Scallan et al., 2011). STEC strains are classified based on the presence of Shiga toxin genes encoded on lambdoid bacteriophages that result in the production of Shiga toxin (O’Brien et al., 1984). Patients with STEC often present with hemorrhagic colitis or bloody diarrhea and in severe cases, hemolytic uremic syndrome (HUS), kidney failure and death can occur (Karmali et al., 1985).

Historically, STEC O157 strains have predominated in clinical infections, causing the greatest number of outbreaks and the most severe clinical outcomes. The incidence of infections caused by strains belonging to other serogroups (i.e., non-O157), however, has increased (Gould et al., 2013). In the years between 2000 and 2015, for instance, Foodborne Disease Active Surveillance Network (FoodNet) reported an increase in the incidence of non-O157 infections from 0.12 to 1.65 per 100,000, while a decrease in O157 incidence from 2.17 to 0.95 per 100,000 was documented (Gould et al., 2013; Crim et al., 2015). The emergence of other serogroups associated with disease has resulted in the classification of the “big-six” serogroups, which represent the predominant non-O157 serogroups and include: O26, O45, O103, O111, O121, and O145 (Brooks et al., 2005). These six serogroups accounted for 83% of non-O157 cases reported to FoodNet from 2000 to 2010 (Gould et al., 2013). Although a wide range of other serogroups are responsible for the remaining infections, less is known about the epidemiology and genetic diversity of these strains relative to O157 STEC.

Multiple methods have been used to examine the genetic diversity of STEC. Multilocus sequence typing (MLST) and pulsed-field gel electrophoresis (PFGE), for instance, allow for the differentiation of isolates, though neither can distinguish closely related isolates with high discriminatory power (Ribot et al., 2006; Sabat et al., 2013). For O157, MLST was found to inadequately differentiate strains (Noller et al., 2003) resulting in the development of more discriminatory schemes such as single nucleotide polymorphism (SNP) genotyping (Zhang et al., 2006; Manning et al., 2008). The recent implementation of whole genome sequencing (WGS) for national surveillance of STEC and other foodborne pathogens, however, has led to the development of new subtyping platforms (Lindsey et al., 2016; Tolar et al., 2019). The preferred subtyping tools recommended by the Centers for Disease Control and Prevention (CDC), which is primarily based on ease of use and potential for data sharing, include the seven gene MLST scheme as well as the core genome (cg) and whole genome (wg) MLST schemes (Ribot et al., 2019). Extraction of high quality (hq) SNPs has also been used to discriminate closely related strains including those that may be associated with outbreaks (Dallman et al., 2015). Although these methods will become the new gold standard for surveillance in the future, there is still a need to utilize discriminatory typing methods that target fewer regions of the genome, particularly for laboratories and nations that lack access to WGS. Additionally, targeted sequencing approaches can be used to examine complex communities for pathogen detection.

In prior studies, clustered regularly interspaced palindromic repeat (CRISPR) loci have been used to characterize and subtype foodborne pathogens like *Salmonella enterica* and *Campylobacter jejuni* (Shariat et al., 2013; Kovanen et al., 2014). This method, however, is not routinely used to examine the population structure of STEC. CRISPR loci, which are important for adaptive immunity, have been found in up to 50% of bacteria (Grissa et al., 2007). These loci comprise a series of direct repeats separated by diverse spacer sequences, which range in size between 21 and 72 bp and are located next to CRISPR associated sequence (*cas*) genes (Jansen et al., 2002; Horvath and Barrangou, 2010). The high degree of diversity in CRISPR-Cas systems is primarily due to the variation within these spacer sequences (Koonin et al., 2017).

It was previously shown that Cas proteins allow for the integration of invasive or foreign DNA fragments as spacers into the CRISPR region (Barrangou et al., 2007; Nuñez et al., 2015). This foreign DNA was found to be derived from phages, plasmids or other mobile genetic elements (Bolotin et al., 2005; Mojica et al., 2005; Pourcel et al., 2005). Transcription of the CRISPR-Cas region results in the assembly of CRISPR RNAs with Cas effector proteins to recognize foreign DNAs (Brouns et al., 2008; Carte et al., 2008; Jackson and Wiedenheft, 2015) for cleavage and degradation (Marraffini and Sontheimer, 2008; Garneau et al., 2010). In *E. coli*, four CRISPR loci have been identified and characterized as CRISPR 1, 2, 3, and 4; these loci are classified as Type I-E or Type I-F depending on the presence of the associated *cas* genes (for a review, see Xue and Sashital, 2019). *E. coli* can also possess CRISPR loci that lack *cas* genes. CRISPR 1 and 2 were defined as having the *iap/cas* and *ygcE/ygcF* genes, respectively, while CRISPR3-4 show little variation within the spacer region (Díez-Villaseñor et al., 2010; Touchon et al., 2011).

Although the impact of CRISPRs on immune function has not been established in *E. coli* in natural conditions, it has been suggested that these systems may have alternative functions (Babu et al., 2011). Nonetheless, the degree of variability within the CRISPR loci were suggested to be useful for subtyping strains (Díez-Villaseñor et al., 2010; Yin et al., 2013). One study of STEC, for example, identified an association between the CRISPR region and the H-antigen (Yin et al., 2013), which is notable given that serotyping based on the O-and H-antigen is the primary classification scheme for STEC. Consequently, the goal of our study was to apply CRISPR subtyping along with MLST, serotyping and virulence gene profiling to characterize non-O157 strains recovered from patients in two geographic locations during an overlapping time period (2000–2006). Examination of this historical strain collection is useful because it allows for the classification of strains from a time when these pathogens were first emerging in the United States. Such documentation is critical as it will enhance our ability to make comparisons to currently circulating strains and evaluate disease patterns caused by specific strain types over time. The use of standardized targeted sequencing methods is helpful for detecting pathogens in complex communities or matrices, examining genetic variation and evolutionary relationships across strain populations, and identifying epidemiological associations with specific genotypes when data are available. Identification of closely related strains and lineages can then be targeted for further characterization using more discriminatory methods such as wgMLST and hqSNP profiling, particularly for sites with access to WGS data and more sophisticated analytical tools.

## Materials and Methods

### Bacterial Strains and Epidemiological Data

The Michigan Department of Health and Human Services (MDHHS) recovered 41 isolates from patient fecal samples during the years 2001–2006 as part of a sentinel surveillance developed specifically for non-O157 STEC (Manning et al., 2007). During an overlapping time period between 2000 and 2005, the Connecticut Department of Public Health (CTDPH) recovered 114 isolates from patient fecal samples as part of the CDC FoodNet. Some epidemiological data were available via the Michigan Disease Surveillance System at the MDHHS and the CTDPH as part of the FoodNet program, though the data were sparse as surveillance for non-O157 STEC was just getting established during this time period. No data were available about the strain source or association with an outbreak.

### DNA Isolation and Whole Genome Sequencing (WGS)

Isolates were grown aerobically overnight in Luria-Bertani broth at 37°C. DNA was isolated using the Wizard^®^ Genomic DNA purification kit and subsequently prepped for sequencing using the Nextera XT kit (Illumina, San Diego, CA, United States) following manufacturer’s instructions. Libraries were sequenced at the MSU Research Technology Support Facility (RTSF) as paired end reads on the Illumina MiSeq platform (2 × 250 reads). *De novo* genome assembly was performed using Spades, 3.10.1 (Bankevich et al., 2012) following trimming and quality checking with Trimmomatic (Bolger et al., 2014) and FastQC (Andrews, 2010), respectively. Multiple k-mers (21, 33, 55, 77, 99, 127) were used and k-mers that passed quality control were cross-assembled to generate the assembly used for downstream analyses. Error correction was performed during the assembly process to minimize the number of mismatches present in the assembled contigs. Sequences were deposited in GenBank^®^ under BioProject PRJNA596289 (SAMN13617411-SAMN13617565).

### Multilocus Sequence Typing (MLST) and *in silico* Analysis of Virulence Genes

Bioinformatic scripts were used to parse results from a local Basic Local Alignment Search Tool (BLAST) (Altschul et al., 1990) with an *E*-value of 0.0001 to ensure specificity of the genes extracted from the assembled genomes as described (Camacho et al., 2009; Cock et al., 2009). The seven gene Whittam MLST typing scheme was utilized, which examines variation in *aspC, clpX, fadD, icdA, lysP, mdh*, and *uidA* as described (Qi et al., 2004). The EcMLST v1.2^1^ was used to assign alleles for each gene to classify strains into sequence types (STs) and define new STs.

Molecular serotyping, which is based on *wzy* and *wzx* (O-antigen lipopolysaccharide) and *fliC* (flagellar H-antigen) genes, was performed using databases hosted by the Center for Genomic Epidemiology^2^. Multiple genes from the National Center for Biotechnology Information (NCBI) were used as references for the Shiga toxin gene variants, *stx1* and *stx2*, as well as the genes encoding intimin (*eae*) and enterohemolysin (*ehxA*) (Supplementary Table S1). To quantify the abundance of prophages embedded in the genomes, Phaster (Arndt et al., 2016) was used to extract prophage-specific sequences, while the Center for Genomic Epidemiology plasmid database was used to quantify the number of plasmids present (Carattoli et al., 2014). Any genes missing from the WGS data were verified using PCR. If a strain was positive for a gene based on PCR, then Sanger sequencing was performed at the MSU RTSF for confirmation.

### CRISPR-Cas Sequence Analysis

Preliminary spacer sequences were identified using CRISPRFinder (Grissa et al., 2008) and verified manually in Geneious (Kearse et al., 2012) to confirm that each spacer sequence was flanked by the respective CRISPR associated genes. Any CRISPR loci that were missing from the genomes were verified by PCR before concluding that a given strain was negative for one or both loci. If these strains were found to be positive for the CRISPR loci based on PCR, then Sanger sequencing was performed for confirmation at the MSU RTSF. PCR primers for CRISPR1 loci were 5′-TGGTGAAGGAGTTGGCGAAGG-3′ and 5′-AAAATGTCCCTCCGCGCTTACG-3′, which annealed *iap* and *cas2* and amplified as described in a prior study (Sheludchenko et al., 2015). CRISPR2 loci were amplified using primers 5′-TACACGCCCTTACGAACACA-3′ and 5′-CCTGGGAAAAGCTTGAGGAT-3′ targeting *ygcE* and *ygcF*, respectively, using the following conditions: 95°C for 3 min followed by 30 cycles of 95°C for 15 s, 69°C for 15 s, and 72°C for 30 s, ending with 72°C for 3 min. A complete list of spacer numbers and sequences is included in Supplementary Table S2.

### Data Analysis

MLST alleles were concatenated and aligned using CLUSTALW, and phylogenetic trees were generated using the Neighbor-joining algorithm with 1000 bootstrap replicates in MEGA7 (Kumar et al., 2016). The complete CRISPR spacer profile was generated using the concatenated sequences of the two CRISPR loci, CRISPR1 and CRISPR2. These CRISPR spacer profiles were converted into a binary code representing the presence and absence of individual spacers. An unweighted pair group method with arithmetic mean (UPGMA) tree was assembled based on the Jaccard similarity index of spacer profiles using Past3 (Hammer et al., 2001). This UPGMA tree was constructed based solely on the presence or absence of specific spacers and not by a direct comparison of concatenated nucleotides. Associations between geographic location, serogroup, epidemiological and molecular data were identified using the Chi-Square (χ^2^) and Mantel-Haenszel Chi-Square test, while the Fisher’s exact test was used for sample sizes less than five. The *t*-test was used to identify differences in means for continuous variables (e.g., the number of CRIPSR spacers). SAS v9.3 (SAS Institute, Cary, NC, United States) was used for the epidemiological analysis; *p* < 0.05 was considered significant and was reported along with the odds ratio (OR) and 95% confidence interval.

## Results

### Characteristics of Cases Infected With Non-O157 STEC by State

From 2000 to 2005, 146 non-O157 STEC infections were reported to the MDHHS (*n* = 32) or the CTDPH (*n* = 114) and were included in this analysis; nine additional Michigan isolates from 2006 were also included. Among the cases, no significant difference in the gender distribution was observed between the two states, though more females were affected in Michigan (64.9%) than Connecticut (54.2%) (Table 1). A significant difference in the age group distribution was observed between states (Mantel-Haenszel χ^2^ *p* = 0.02). Most Michigan cases were between 11 and 29 (32.4%) or 30 and 64 years (40.5%); only 6 (16.2%) cases were less than 10 years of age. Connecticut had a similar proportion of cases between 11 and 29 years (36.4%) but the number of cases under the age of 10 was greater (35.5%) than in Michigan. Both states had a similar proportion of elderly cases over the age of 65 years (Michigan: 10.8%, Connecticut: 9.3%).

**TABLE 1:** Comparison of demographics and clinical outcomes among non-O157 STEC cases from Michigan and Connecticut between 2001 and 2006.

Characteristic	Total no. Michigan	No (%) Michigan	Total no. Connecticut	No (%) Connecticut	Odds Ratio (95% CI^†^)	*p*-value^‡^
**Demographics**						
Sex	37		107			
Male		13 (35.1)		49 (45.8)	1.5 (0.72, 3.38)	0.26
Female		24 (64.9)		58 (54.2)		
Age in years	37		107			
0–10		6 (16.2)		38 (35.5)	0.5 (0.17, 1.51)	0.22
11–29		12 (32.4) 15 (40.6)		39 (36.5) 20 (18.7)	1.0	–
30–64					2.4 (0.96, 6.18)	0.06
≥65		4 (10.8)		10 (9.4)	–	0.73
**Clinical Outcomes**						
Abdominal pain/cramps	26		62			1.0
No		5 (19.2)		12 (19.4)	1.0 (0.32, 3.22)	
Yes		21 (80.8)		50 (80.7)		
Any bloody diarrhea	27		66			
No		8 (29.6)		32 (48.5)	2.2 (0.86, 5.82)	0.096
Yes		19 (70.4)		34 (51.5)		
Hospitalization	27		107			
No		13 (48.5)		95 (88.8)	8.5 (3.25,22.37)	<0.0001
Yes		14 (51.9)		12 (11.2)		

*Number of isolates may not add up to the total (*n* = 155) for some variables due to missing data in case reports. ^†^95% confidence interval for the odds ratio (OR). ^‡^*p*-value for statistical significance calculated using the Likelihood Ratio Chi-Square or Fisher’s exact test for variables with *n*<5 in at least on cell; ORs were not calculated for variables with <5 per cell. The reference group for age was the 11–29 age group.*

Among the 134 cases with data available, differences were observed in symptom reports between the two states (Table 1). A greater proportion of Michigan cases were hospitalized (*n* = 14; 51.9%) compared to Connecticut cases (*n* = 12; 11.2%) (*p* < 0.0001). Among the 26 hospitalized cases, those between 19 and 64 years of age were significantly more likely to be hospitalized (57.7%) compared to those under the age of 18 (23.1%) and over the age of 65 (19.2%) combined [Odds ratio (OR): 3.5; 95% Confidence interval (CI): 1.46, 8.59]. Gender was not significantly associated with hospitalization, though more females (*n* = 17; 65.4%) than males (*n* = 9; 34.6%) were hospitalized. Among a subset of 93 cases with data available, no significant difference was observed in the proportion of cases reporting bloody diarrhea between states although slightly more Michigan (70.4%) cases were affected than Connecticut (51.5%) cases (*p* = 0.09). In all, only one Michigan case presented with HUS, which was caused by a *stx1-*positive strain belonging to serotype O103:H2.

### Molecular Characteristics and Association With Clinical Outcomes

**FIGURE 1:**
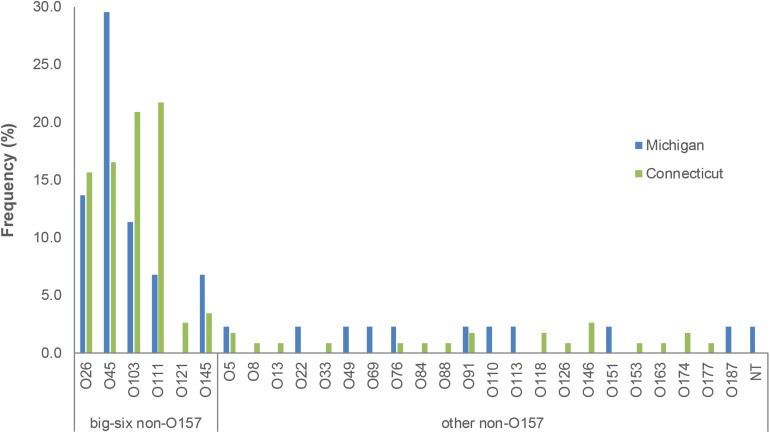
Prevalence of serogroups detected in Michigan and Connecticut, 2001–2006. NT, non-typeable.

A total of 29 serogroups were recovered from the two states; 8 (27.6%) and 13 (44.8%) of these 29 serogroups were found solely in Michigan and Connecticut, respectively, while the remaining 8 serogroups were found in both locations (Figure 1). Among these eight serogroups, most (*n* = 5; 62.5%) belonged to the predominant “big-six” serogroups, though O121 was not detected in Michigan during this time period. The remaining three serogroups found in both locations were O5, O76, and O91. In addition to the lack of O121 in Michigan, other differences in the distribution of some serogroups were observed between states. O45 strains, for instance, were significantly more common in Michigan (95% CI: 1.02, 5.28) than Connecticut. Although the frequency of O111 was eight times higher in Connecticut (89.3%) than Michigan (10.7%), this difference was not statistically significant (Fisher’s exact test *p* = 0.056), which could be due to the small sample size. No differences were observed in the distribution of O26, O103, O121, and O145 by state. Similarly, the virulence gene profiles between the two states were similar based on the presence of *stx, eaeA* or *ehxA*. The presence of *stx1* alone and in combination with *stx2* were the two most common toxin gene profiles in both states, comprising 87.8% of Michigan strains and 90.3% of Connecticut strains.

Among all infections in both states, no significant difference in gender was observed (Table 2). Cases infected with big-six serogroups from Michigan, however, were significantly more likely to be over 30 years of age relative to Connecticut cases (OR: 2.7; 95% CI: 1.08, 6.56). Cases with big-six STEC infections were also significantly more likely to report abdominal cramps and diarrhea with blood relative to cases infected with other non-O157 strains. Differences were also observed in the type of symptoms reported by cases from each state. Connecticut cases with big-six infections, for instance, were significantly more likely to report diarrhea with blood (Fisher’s exact test *p* = 0.03) compared to the big-six cases in Michigan. By contrast, cases with big-six infections in Michigan were significantly more likely to be hospitalized compared to the Connecticut cases with big-six infections (OR: 6.3; 95% CI: 2.18, 18.41). No difference was observed in the number of cases reporting abdominal cramping by state (*p* = 0.80).

**TABLE 2:** Demographic, molecular profiles and clinical outcomes associated with big-six non-O157 serogroups and all other non-O157 serogroups from cases in Michigan and Connecticut combined.

Characteristic	Total no. non-O157 big-six	No (%) non-O157 big-six	Total no. non-O157 other	No (%) non-O157 other	OR (95% CI)^†^	*p*-value^†^
**Demographics**						
State	123		32			
Michigan		30(24.4)		11(34.4)	0.6(0.27, 1.42)	0.26
Connecticut		93(75.6)		21(65.6)		
Sex	114		30			
Male		51(44.7)		11(36.7)	1.4(0.61, 3.20)	0.43
Female		63(55.3)		19(63.3)		
Age in years	114		30			
0–10		34(29.8)		10(33.3)	1.9(0.51, 6.94)	0.48
11–29		43(37.7)		8(26.7)	3.0(0.79, 11.27)	0.13
30–64		28(24.6)		7(23.3)	2.2(0.56, 8.76)	0.29
≥65		9(7.9)		5(16.7)	1.0	–
**Virulence Genes**						
Shiga toxin	123		32			
*stx1* only		106(86.1)		16(50.0)	10.6(3.09, 36.34)	<0.0001
*stx2* only		7(5.7)		9(28.1)	0.5(0.14, 2.17)	0.39
*stx1/stx2*		10(8.1)		7(21.9)	1.0	–
*eaeA*	123		32			
No		3(2.4)		19 (59.4)	58.5 (15.22, 224.49)	<0.0001
Yes		120(97.6)		13 (40.6)		
*ehxA*	123		32			
No		4(3.2)		10(31.2)	13.5(3.89, 49.99)	<0.0001
Yes		119(96.8)		22(68.8)		
**Clinical Outcomes**						
Abdominalpain/cramps	71		17			
No		10(14.1)		7(41.2)		
Yes		61(85.9)		10(58.8)	4.3(1.32, 13.82)	0.01
Any bloody diarrhea	75		18			
No		27(36.0)		5(27.8)	4.6(1.49, 14.37)	0.005
Yes		48(64.0)		13(72.2)		
Hospitalization	108		26		–	0.78
No		86(79.6)		22(84.6)		
Yes		22(20.4)		4(15.4)		

*Total isolates for each variable examined may not add up to the total (*n* = 155) due to missing epidemiological information in case reports. ^†^ 95% confidence interval (CI) for the odds ratio (OR) reported. ^‡^*p*-value for statistical significance calculated using Chi-Square test or Fisher’s exact test for variables with *n*<5 in at least on cell; ORs were not calculated for variables with <5 per cell.*

Stratifying by serogroup identified several associations as well, particularly when the big-six serogroups were analyzed individually and compared to the other non-O157 serogroups (Table 3). Most notably, the O45 cases were significantly more likely to be hospitalized (OR: 2.6; 95% CI: 1.02, 6.87) compared to cases infected with all other serogroups. In addition, children younger than 18 years old were significantly more likely to have O111 STEC infections (OR: 4.2; 95% CI: 1.48, 11.95), while cases over 19 years of age were significantly more likely to have O45 infections (OR: 3.3; 95% CI: 1.40, 7.96) compared to all other non-O157 serogroups. When stratified by state, 87.0% of the O111 cases in Connecticut occurred in children under 18 years of age (Mantel-Haenszel χ^2^ *p* = 0.03). Although a difference by sex was observed as males were more likely to have O111 infections than females, this difference was not statistically significant (*p* = 0.06).

**TABLE 3:** Demographics, molecular profiles and clinical outcomes associated with big-six non-O157 serogroups from cases in both Michigan and Connecticut relative to infection with other non-O157 serogroups.

Characteristic*	O26 (*n* = 24) No (%)	O45 (*n* = 32) No (%)	O103 (*n* = 29) No (%)	O111 (*n* = 28) No (%)	O121 (*n* = 3) No (%)	O145 (*n* = 7) No (%)	Other (*n* = 32) No (%)	χ^2‡^	*p*^‡^
**Demographics**									
State									
Michigan	6(25.0)	13(40.6)	5(17.2)	3(10.7)	0(0.0)	3(42.9)	11(34.4)	1.89	0.17
Connecticut	18(75.0)	19(59.4)	24(82.8)	25(89.3)	3(100.0)	4(57.1)	21(65.6)		
Sex									
Male	6 (27.3)	14(48.3)	12(42.9)	15(60.0)	0(0.0)	4(57.1)	11(36.7)	0.001	0.97
Female	16(72.7)	15(51.7)	16(57.1)	10(40.0)	3(100.0)	3(42.9)	19(63.3)		
Age in years									
0–10	8 (36.4)	4 (13.8)	6 (21.4)	12(48.0)	1(33.3)	3(42.9)	10(33.3)		
11–29	6 (27.3)	12(41.4)	14(50.0)	8(32.0)	0(0.0)	3(42.9)	8(26.7)	2.31	0.13
30–64	5 (22.7)	10(34.5)	8(28.6)	3(12.0)	1(33.3)	1(14.2)	7(23.3)		
≥65	3 (13.6)	3(10.3)	0(0.0)	2(8.0)	1(33.3)	0(0.0)	5(16.7)		
**Virulence Factors**									
Shiga toxin									
*stx1* only	24(100.0)	32(100.0)	29(100.0)	20(71.4)	0(0.0)	1(14.3)	16(51.6)	0.16	0.69
*stx2* only				0(0.0)	3(100.0)	4(57.1)	9(29.0)		
*stx1*/*stx2*	0(0.0) 0(0.0)	0(0.0) 0(0.0)	0(0.0) 0(0.0)	8(28.6)	0(0.0)	2(28.6)	6(19.4)		
*eaeA*									
No	2(8.3)	0(0.0)	1(3.4)	0(0.0)	0(0.0)	0(0.0)	19(59.4)	25.68	<0.0001
Yes	22(91.7)	32(100.0)	28(96.6)	28(100.0)	3(100.0)	7(100.0)	13(40.6)		
*ehxA*									
No	2(8.3)	1(3.1)	1(3.4)	0(0.0)	0(0.0)	0(0.0)	10(31.3)	9.86	0.0017
Yes	22(91.7)	31(96.9)	28(96.6)	28(100.0)	3(100.0)	7(100.0)	22(68.7)		
**Clinical Outcomes**									
Abdominal pain/cramps									
No	4(30.8)	2(10.0)	1(5.9)	3(20.0)	0(0.0)	0(0.0)	7(41.2)	1.26	0.26
Yes	9(69.2)	18(90.0)	16(94.1)	12(80.0)	1(100.0)	5(100.0)	10(58.8)		
Diarrhea with blood									
No	5(38.5)	6(28.6)	8(42.1)	7(43.8)	0(0.0)	1(20.0)	13(72.2)	3.38	0.07
Yes	8(61.5)	15(71.3)	11(57.9)	9(56.2)	1(100.0)	4(80.0)	5(27.8)		
Case Hospitalization									
No	16(80.0)	18(66.7)	22(84.6)	23(92.0)	3(100.0)	4(57.1)	22(84.6)	0.005	0.94
Yes	4(20.0)	9(33.3)	4(15.4)	2(8.0)	0(0.0)	3(42.9)	4(15.4)		

*^∗^Total isolates for each variable examined may not add up to the total per column due to missing data in case reports. ^‡^*p*-value for statistical significance calculated using Mantel-Haenszel Chi-Square (df = 1) for the association between each characteristic and serogroup.*

**FIGURE 2:**
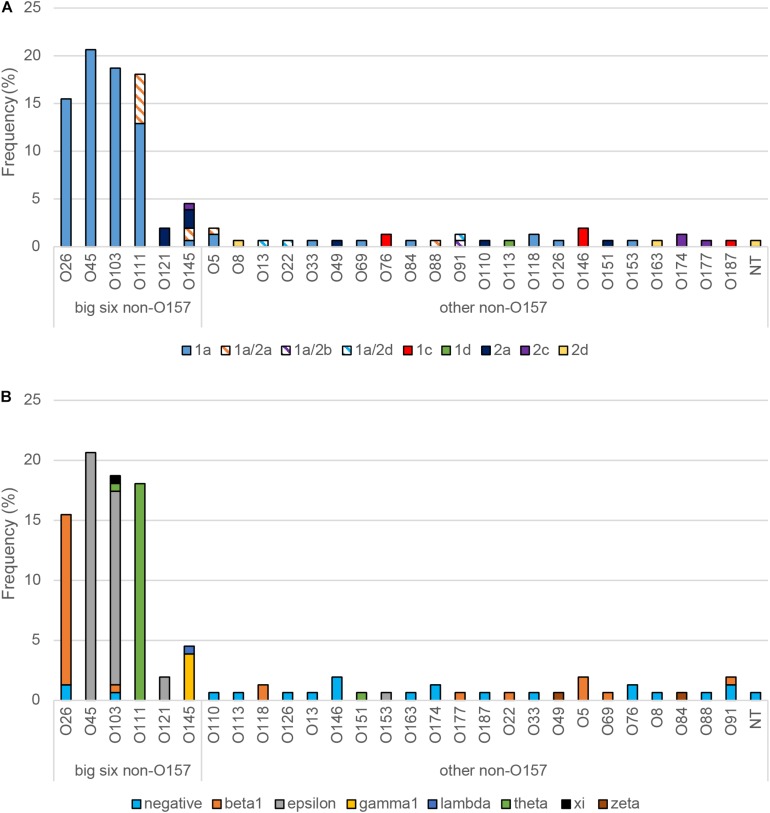
Distribution and frequency of virulence gene alleles among non-O157 Shiga toxin-producing *Escherichia coli* isolates recovered from Michigan and Connecticut. The frequency of **(A)** Shiga toxin (*stx*) gene alleles and **(B)** intimin (*eae*) gene variants are shown. NT, non-typeable.

When the virulence gene profiles were examined, the big-six serogroups were more likely to have *stx1* (OR: 6.5; 95% CI: 2.19, 19.18) alone or in combination with *stx2*, compared to all other serogroups (Table 3). Similarly, the big-six serogroups had a significantly higher frequency of *eaeA* (OR: 58.5; 95% CI: 15.22, 224.49) and *ehxA* (OR: 13.5; 95% CI: 3.89, 49.99) compared to all other serogroups. Those isolates representing serogroups O26, O45, O103, and O111 had either *stx1* (*n* = 105) or *stx1/stx2* (*n* = 8), while the three O121 isolates had *stx2a* only (Figure 2A). Among the big-six serogroups, however, the O145 isolates were the most diverse containing multiple *stx* profiles. Comparatively, a wider range of *stx* variants/profiles (*n* = 9) were observed among the other non-O157 serogroups, further highlighting the heterogeneity of the non-O157 strain population. Although the *eaeA* gene profiles were relatively homogeneous within a serogroup, seven different *eaeA* variants were identified among all 155 isolates (Figure 2B). Moreover, many (59.4%) of the non-O157 strains outside of the big-six group were negative for *eaeA*.

### Genetic Diversity of Non-O157 STEC and Association With Disease

**FIGURE 3:**
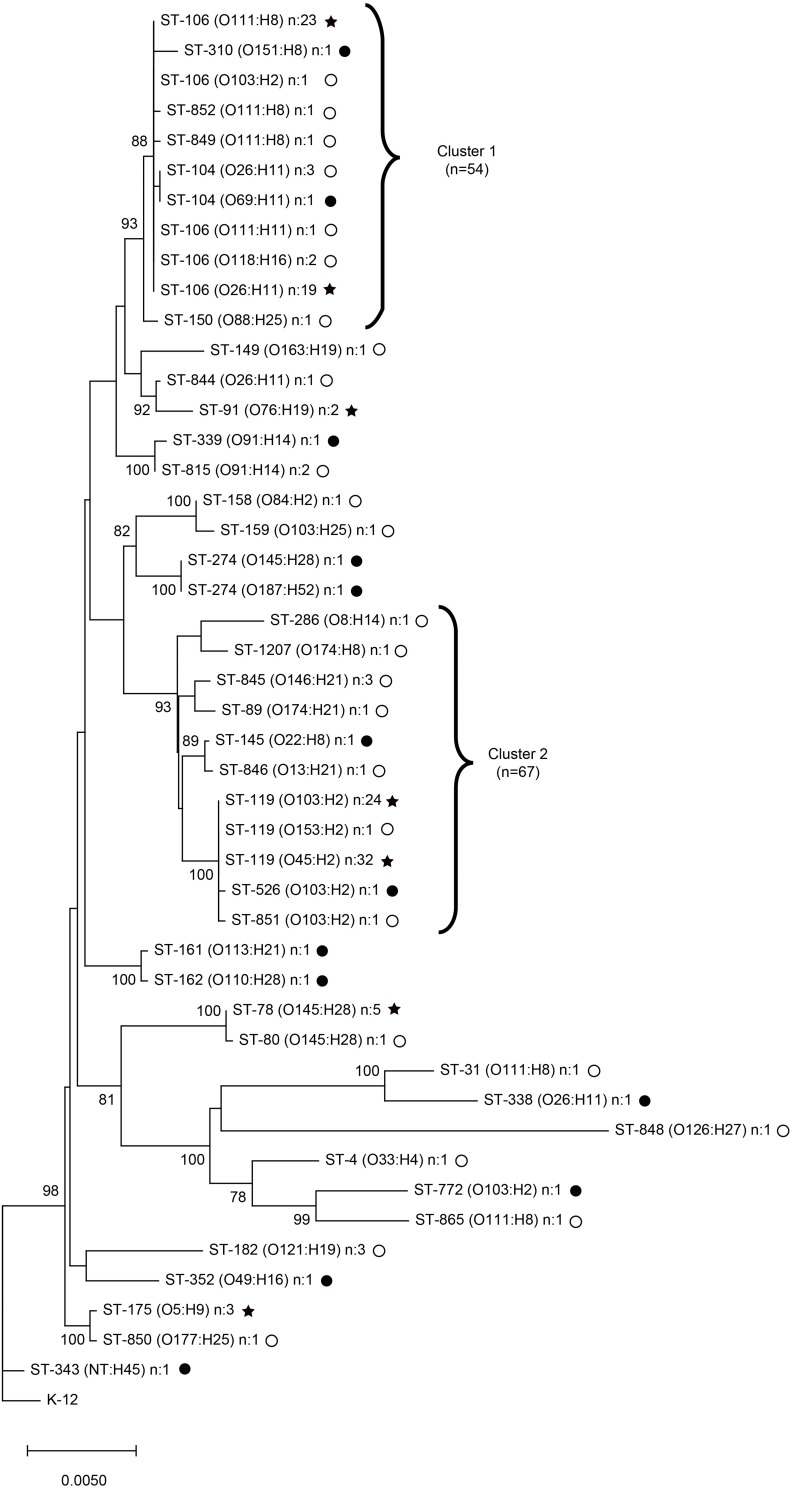
Neighbor-joining phylogeny constructed using seven MLST genes for 155 clinical non-O157 Shiga toxin-producing *Escherichia coli* (STEC) isolates from Michigan (*n* = 44, black circles) and Connecticut (*n* = 111, open circles) with 1,000 bootstrap replicates. Clusters 1 and 2 represent sequence types (STs) that grouped together with >90% bootstrap values. STs shared across the two geographic locations are indicated with black stars. NT, non-typeable.

MLST was utilized to examine the genetic diversity of the STEC strains isolated from both states (Figure 3). A total of 38 STs were identified in all; 17 STs were recovered in Michigan and 27 STs were collected in Connecticut. Six of the STs were shared and found in both locations. The shared STs comprised 75.5% of the cases in the two states, with ST-106 [MI: *n* = 8 (19.5%); CT: *n* = 38 (33.3%)] and ST-119 [MI: *n* = 16 (39.0%); CT: *n* = 41 (36.0%)] predominating. One isolate from Connecticut was classified as a new ST with a unique allele profile; it was designated as ST-1207.

A neighbor-joining phylogenetic tree with bootstrapping (*n* = 1000) grouped the strains into two clusters with greater than 90% bootstrap support. The first cluster, Cluster 1, contains STs 104, 106, 150, 310, 849, and 852, while Cluster 2 contains STs 89, 119, 145, 286, 526, 845, 846, 851, and 1207 (Figure 3). All strains not grouping within these two clusters were considered as the “other” group for the subsequent epidemiological analyses. Strains within Cluster 1 contained eight different serotypes including O88:H25, O26:H11, O118:H16, O111:H11, O69:H11, O111:H8, O103:H2, and O151:H8, whereas Cluster 2 included serotypes O103:H2, O153:H2, O45:H2, O22:H8, O13:H21, O146:H21, O174:H21, O8:H14, and O174:H8. Only one serotype, O103:H2, was found in both Clusters 1 and 2 as well as a smaller unrelated cluster. Multiple serotypes are represented by genetically unrelated STs and were found across different branches of the tree. O103:H2 strains, for example, represented STs 772, 106, 851, 526, and 119, while O26:H11 strains comprised STs 338, 104, 106, and 844. Notably, strains of the same serogroup belonged to multiple STs and clustered separately on different branches of the phylogenetic tree.

None of the *stx* combinations were significantly different between the clusters identified by MLST. Variants *ehxA*-*F* were significantly more common in Cluster 2 (OR: 31.5; 95% CI: 12.28, 80.83), while *ehxA*-C was more common in Cluster 1 (Fisher’s exact test *p* < 0.0001) relative to all other isolates. Strains with other *ehxA* variants or that lacked *ehxA* altogether were not associated with a specific cluster. Similarly, the *eaeA* variants, beta (OR: 11.6; 95% CI: 4.5, 29.5) and epsilon (Fisher’s exact test *p* < 0.0001), were the only two variants found in Cluster 1. The epsilon variant predominated (86.6%) and was significantly more common in isolates from Cluster 2 relative to all other clusters (Fisher’s exact test *p* < 0.0001). Strains harboring other *eaeA* variants or that lacked *eaeA* were located throughout the phylogenetic tree with the exception of *eaeA*-xi, which was only found in a O103:H2, ST-119 strain grouping within Cluster 2.

No clustering of strains was observed by state; however, age was significantly associated with cluster designation. Cases with non-O157 STEC belonging to Cluster 1 (*n* = 35; 70.0%) were significantly more likely to be young, or less than 18 years of age, compared to the 27 (44.3%) strains belonging to Cluster 2 (OR: 2.9; 95% CI: 1.34, 6.46) or all other Clusters (*n* = 16; 48.5%; OR: 2.5; 95% CI: 1.0, 6.17). No associations were identified between Cluster and more severe clinical outcomes like hospitalization and presence of blood in the stool.

### CRISPR Profiling and Phylogenetic Analysis

Clustered regularly interspaced repeat 1 and CRISPR2 loci were identified in 149 of the 155 strains. Because the CRISPR2 loci were not detected in sequences from six strains, those strains were excluded from the analysis. Two of these strains, TW14929 (O103:H2) and TW10122 (O26:H11), were missing the CRISPR2 loci entirely and both lacked CRISPR spacers and repeats in the region between *ygcE* and *ygcF*. Another strain, TW14904 (O111:H8), had an interrupted CRISPR2 locus with a potential insertion element that lacked any spacers or repeat sequences.

The 149 strains examined had a range of six to 49 unique CRISPR spacer sequences from both the CRISPR1 and CRISPR2 loci (Supplementary Table S3). The CRISPR1 loci had between one and 30 spacers, while CRISPR2 had between zero and 21 spacers. A total of 361 unique spacers were identified that grouped into 80 different CRISPR profiles; these profiles are represented by the numbers and sequences of each spacer. The presence of spacer 56 (*n* = 62), 231 (*n* = 118), and 317 (*n* = 88) was detected in multiple strains regardless of serogroup or ST. In all, each strain had an average of 14 spacers and 13 (8.7%) strains had more than 20. No difference was observed in the average number of spacers by MLST cluster, though strains with similar spacer profiles also grouped together in the MLST phylogeny. Most strains belonging to Cluster 1 (*n* = 36; 69.2%) had between 11 and 20 spacers, while 53.0% (*n* = 35) of the Cluster 2 strains had 11–20 spacers.

When stratifying by serogroup, significant differences in spacer content were observed (Mantel-Haenszel χ^2^ *p* < 0.0001) with the big-six serogroups having fewer spacers than all other serogroups combined. The average number of spacers was 12.8 for the 120 strains belonging to the big-six serogroups compared to 20.2 for the 29 strains representing other serogroups (*t*-test *p* = 0.0006).

**FIGURE 4:**
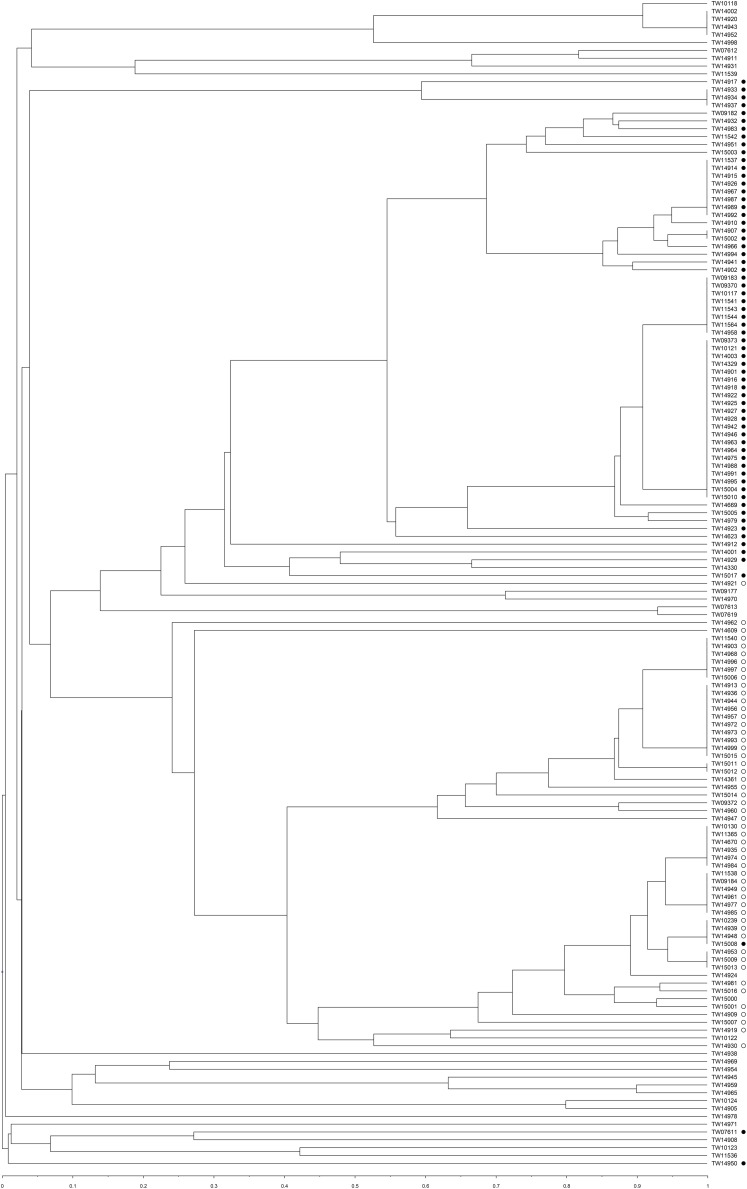
Unweighted pair group method with arithmetic averages (UPGMA) clustered using a Jaccard similarity index to compare the spacer patterns of the CRISPR profiles of 149 total isolates from Michigan (*n* = 40) and Connecticut (*n* = 109). Strains belonging to Cluster 1 are indicated with black circles and Cluster 2 strains are indicated with open circles.

Concatenation of the spacers detected in both CRISPR loci enabled an assessment of relatedness using a UPGMA analysis (Figure 4). Overall, the CRISPR profiles of the strains clustered similarly to those identified by MLST regardless of serogroup (both O and H-type) and source.

Since twice as many CRISPR profiles were identified compared to STs in the MLST analysis, the Simpson’s diversity index was calculated for each genotyping method. The discriminatory power of MLST and CRISPR profiling was 0.76 and 0.96, respectively, while the power for both methods combined was 0.97. Nonetheless, several discrepancies were observed between the methods. For example, TW15008, a ST-119 serotype O103:H2 strain, belongs to Cluster 2 in the MLST phylogeny but groups together with other Cluster 1 strains in the UPGMA tree based on CRISPR spacer content (Figure 4). Another O103:H2 strain (TW14919) was classified as ST-106; it grouped within Cluster 1 using MLST and had a CRISPR profile similar to other strains within Cluster 1, which was distinct from other O103:H2 strains of Cluster 2.

### CRISPR Spacer Content Indicative of Phage and Plasmid Transfer

Only 5.5% (*n* = 20) of all 361 spacers belonged to known or putative phages and plasmids with at least 3 nucleotide differences when screened using BLAST against the NCBI database. Spacers that matched to CRISPR spacers found in other organisms including *Shigella sonnei*, were not noted. Spacer 356 was of interest because 30 of the 32 nucleotides matched the common *E. coli* O157 T7 typing phage (Cowley et al., 2015). Although spacers 56, 231, and 317 were common to strains regardless of genetic relatedness, these spacers did not match any published phage or plasmid sequences. The total horizontal gene transfer value was assigned to each genome by adding together the number of phages and plasmids present in the genomes. Strains with ≥20 spacers had a significantly higher number of horizontally acquired elements (total plasmids and phages ≥8) (OR: 4.9; 95% CI: 1.50, 16.36) when compared to strains with a lower (<20) spacer content.

## Discussion

Although non-O157 STEC infections have been steadily increasing in the United States since 2000 (Gould et al., 2013; Centers for Disease Control and Prevention, 2017), little is known about the molecular epidemiology and genetic diversity of these pathogens in different geographic locations, particularly for older strain sets. Through this analysis, we have shown that a wide range of strain types are linked to human infection in two states and that strains representing one of the six (“big-six”) most abundant serogroups predominated in each. Variation in epidemiological factors among cases from each state was also observed as well as variation in the molecular characteristics of the STEC populations.

In all, a greater number of cases were detected in Connecticut compared to Michigan over the same time period, which could be due to differences in surveillance activities. Connecticut participated in the FoodNet active surveillance system, while Michigan utilized a sentinel surveillance system established by the MDHHS (Manning et al., 2007). The age distribution also varied among cases from each state. Most STEC cases reported by FoodNet occur in young children or the elderly (Gould et al., 2013), which was similar to the age distribution in Connecticut. In Michigan, however, most cases were between 19 and 64 years of age. In addition to surveillance methods, such differences could be due to varying environmental factors, behavioral practices, or occupational risks. Indeed, Michigan has a larger number of dairy cattle farms (Economic Research Service United States Department of Agriculture, 2006) and prior studies have linked high cattle densities to STEC infections caused by specific STEC serogroups (Frank et al., 2008; Friesema et al., 2010). Cases in Michigan were also more likely to be hospitalized compared to cases from Connecticut. Because gastrointestinal infections are underreported and Michigan was not participating in active surveillance, cases with less severe infections may have been less likely to be screened for non-O157 STEC relative to hospitalized cases. Higher hospitalization rates in Michigan could also be due to a lower threshold for hospital admission or may indicate variation in virulence of the STEC strains recovered from each state.

Each big-six serogroup with the exception of O121, was found in both states as were strains belonging to serogroups O5, O76, and O91, which have been linked to human infections in Europe (Beutin et al., 2004; Trotter et al., 2011; Messens et al., 2015). Although O121 strains were missing from the Michigan population during this time period, we cannot definitively state that they were absent given the limitations associated with the use of a sentinel surveillance system. Hence, we cannot rule out the possibility that surveillance methods were biased to detect specific serogroups over others and that the findings may not be generalizable to the whole population. By contrast, serogroup O91 is among the most frequently isolated serogroups in foods and human infections in Europe (Trotter et al., 2011). The close proximity of Connecticut and Michigan to international airports or borders may indicate that some of these infections were travel-associated as associations between infection with O111, O103, and O26 strains have been linked to international travel in prior studies (Lathrop et al., 2009; Gould et al., 2013; Tseng et al., 2016). These data, however, were not available for cases in either state and therefore, future studies are needed to establish relationships between travel and risk of infection with specific strain types.

The MLST-based phylogenetic analysis failed to identify clustering of strains by geographic location, with most (75.5%) belonging to STs that were detected in both states. Strains belonging to STs 106 and 119 were found in highest frequency, though neither were comprised of strains representing only one serogroup. ST-106, for instance, was primarily composed of O26 and O111 serogroups, while ST-119 had strains of serogroups O45 and O103. Another study, which used a different MLST scheme, indicated that the clustering of O26 and O111 strains could be due a lateral gene transfer event of the *rfb*-like region similar to that which occurred in the emergence of O157 (Feng et al., 1998; Eichhorn et al., 2015). In fact, a wide range of serogroups clustered together with the big-six serogroups into two main clusters. Examination of these clusters found Cluster 1 to be significantly more common among patients younger than 18 years old. This association, however, may be due to the presence of serogroup O111:H8, which was also significantly associated with young age and composed a large percentage (42.6%) of Cluster 1 strains. The genetic relatedness of strains with different serogroups strongly suggests that serogroup alone should not be an indication of disease outcome. The presence of different serogroups in multiple genetically unrelated branches on the tree, such as O103:H2 strains representing ST-106, 119, 526, 772, and 851, further highlights the genetic diversity within a single serogroup. Similarly, multiple strains linked to different disease outcomes were classified as having the same serogroup and ST, thereby indicating that more refined analytical approaches should be used to determine how such strains differ across populations and impact virulence. Such approaches could include the use of cgMLST and wgMLST schemes, which have better discriminatory power and can identify the most closely related strains among those with similar profiles. Extracting sequences specific for critical STEC virulence genes such as *stx*, *eae*, and *ehxA*, can also enhance discriminatory power, though it is important to note that many of these genes reside on mobile DNA elements. Although we considered using more comprehensive genomic typing schemes, these approaches are too discriminatory for our purpose, which was to make comparisons to strains isolated in two regions and not to identify strains that are identical or part of multistate outbreaks. Unfortunately, this older set of strains lacked detailed epidemiological data and therefore, we were limited in our ability to draw conclusions about outbreaks and other associations.

The added use of CRISPR loci analysis to MLST enhanced the discriminatory power from 0.76 for MLST alone to 0.97. Application of CRISPR spacer analysis has been previously used to discriminate *S. enterica* and *C. jejuni* outbreak isolates (Kovanen et al., 2014; Shariat et al., 2015). Amplification of two CRISPR regions, MLST loci, and serogroup genes is less expensive and time consuming than PFGE and reveal more about the genetic relatedness and variation that is present among strains (Ribot et al., 2006). In this study, use of MLST identified a O103:H2 strain (TW15008) that clustered with ST-119, while an additional assessment of spacer sequences from the two concatenated CRISPR loci demonstrated that this strain was more similar to strains clustering with ST-106. This finding suggests that a potential evolutionary event may have occurred that would have been missed if only examining the MLST profile. Indeed, the use of CRISPR spacers has been previously used to examine the evolutionary divergence of O55:H7 to O157:H7 (Yin et al., 2013). Even though our data also suggests that some spacer sequences may be linked to specific clusters, a larger sample of strains would need be examined to identify whether specific spacers are associated with specific lineages and clinical outcomes.

Analysis of the CRISPR spacers that were detected in this set of strains identified 5.5% of the spacers to originate from known phages or plasmids; this finding is similar to what has been reported in other studies that have examined spacer content (Mojica et al., 2005; Yin et al., 2013). While the putative function of the CRISPR loci is to provide adaptive immunity, in laboratory conditions, STEC is not provided with immunity when subjected to plasmids or phages that have corresponding spacers in the CRISPR loci (Edgar and Qimron, 2010; Mojica and Díez-Villaseñor, 2010; Touchon et al., 2012). However, the number of spacers that are present in the CRISPR loci may be indicative of a strain living in an environment that is subject to high rates of horizontal gene transfer. Strains with a higher number of CRISPR spacers were significantly more likely to encode a higher number of plasmids or phages in their genome. This finding provides further support for STEC having an active CRISPR loci in specific conditions outside of the laboratory. An alternative explanation is that a recent event could have turned off the CRISPR loci, thereby enhancing uptake of plasmids and phages without the foreign DNA being targeted by the CRISPR system.

Overall, this study enhances understanding of the genetic composition and relatedness of clinical non-O157 STEC strains in two different geographic locations during a time when surveillance efforts for non-O157 strains were commencing. It is important to note, however, that epidemiological information was missing for some cases and that some of the differences identified between the two states could be due to the use of different surveillance systems. Michigan non-O157 STEC frequencies may have also been underestimated given the use of a sentinel system; hence the associations require confirmation in future studies with larger numbers of strains. Nonetheless, the ability to subtype strains by extracting informative sequences such as CRISPR spacers, virulence genes, and MLST loci from WGS data can be used to detect and classify strains in complex communities and is most helpful for laboratories that lack access to or the ability to analyze WGS data.

## Data Availability Statement

Sequences were deposited in GenBank^®^ under BioProjectID PRJNA596289 with Biosample IDs SAMN13617411 to SAMN13617565.

## Ethics Statement

The studies involving human participants were reviewed and approved by The Institutional Review Boards at Michigan State University (#10-736SM), the Michigan Department of Health and Human Services (#842-PHALAB), and the Connecticut Department of Public Health. Written informed consent from the participants’ legal guardian/next of kin was not required to participate in this study in accordance with the national legislation and the institutional requirements.

## Author Contributions

HB, SM, and JR designed the study in Michigan. QP and JF designed the study in Connecticut. RM and QP organized the samples and extracted the epidemiological data. HB developed and performed the bioinformatics analysis. HB and SM analyzed the data and drafted the manuscript. All authors contributed and approved the manuscript content.

## Conflict of Interest

The authors declare that the research was conducted in the absence of any commercial or financial relationships that could be construed as a potential conflict of interest.

## Footnotes

1 www.shigatox.net

2 www.genomicepidemiology.org/
