# Supplementary material for: Genetic Diversity of Non-O157 Shiga Toxin-Producing Escherichia coli Recovered From Patients in Michigan and Connecticut
Source: Front Microbiol. 2020 Mar 31;11:529. doi: 10.3389/fmicb.2020.00529 (PMC7145412; doi:10.3389/fmicb.2020.00529)
Supplement: Supplementary file 1 [file Data_Sheet_1.PDF]

Table S1. Accession numbers linked to each gene variant analyzed in the study.

Gene	Gene variant	National Center for Biotechnology Information (NCBI) Accession number
Shiga toxin gene (*stx*)	*stx1a*	M19473.1
	stx1a	AM230662.1
	stx1c	Z36901.1
	stx1c	AB048237.1
	stx1d	AY170851.1
	stx2a	X07865.1
	stx2a	EF441609.1
	stx2b	AF043627.1
	stx2b	AB048226.1
	stx2c	M59432.1
	stx2c	EU086525.1
	stx2d	FM998855.1
	stx2d	DQ059012.1
	stx2e	X81418.1
	stx2e	AY332411.1
	stx2f	AJ010730.1
	stx2f	AB472687.1
	stx2g	AY286000.1
	stx2g	AB048227.1
Enterohemolysin (*ehxA*)	*ehxA-A*	AY258503.2
	ehxA-B	AP018692.1
	ehxA-C	AP010954.1
	ehxA-D	EF204927.1
	ehxA-E	EF204923.1
	ehxA-F	AP010959.1

Gene	Gene variant	NCBI Accession Number
Intimin (*eaeA*)	alpha1	AF022236.1
	alpha2	AF530555.1
	beta1	MK761162.1
	beta2	AJ715407.1
	delta	AJ875027.1
	kappa	AJ308552.1
	gamma	CP034384.1
	theta	AF449418.1
	epsilon	AF116899.1
	epsilon2	DQ523614.1
	zeta	AJ271407.1
	eta	AJ308550.1
	eta2	AJ876652.1
	jota	DQ523601.1
	jota2	AF530553.1
	lambda	AJ715409.1
	mu	AJ705049.1
	nu	AJ705050.1
	xi	AJ705051.1
