# Supplementary material for: Genetic Diversity of Non-O157 Shiga Toxin-Producing Escherichia coli Recovered From Patients in Michigan and Connecticut
Source: Front Microbiol. 2020 Mar 31;11:529. doi: 10.3389/fmicb.2020.00529 (PMC7145412; doi:10.3389/fmicb.2020.00529)
Supplement: Supplementary file 2 [file Data_Sheet_2.PDF]

Table S2. CRISPR spacers numbers, sequence data and putative origin for 361 spacers that were identified in the study. Each spacer number corresponds to a specific DNA sequence.

Spacer	Spacer sequence	Comments
spacer 1	AACTCTCACCGGAGCAGAAAAAAAGAAACCAG	
spacer 2	GCGGACGGCAAAACAATCGGGAGTCTGGACAA	
spacer 3	ACTATCGGCGCTGTTCAGTTGGGACAGAAACA	
spacer 4	CCCGTTGAAGATCTGGACGCGTGGGGAAAAGC	
spacer 5	GGAGGATGTTCGGTCCCGGAACTAAACCGGCA	hypothetical protein in putative phage region
spacer 6	CCGTCAGTAAACCCGCTGGCGGGTACTGGATAGTGTTCCCCGCGCCAAAAAAAATT	
spacer 7	CGTGTCTGCCGCTGCTGAGCTGTGCGGGGATA	
spacer 8	GCGCTCTCCTGAACCTCTTTCAGTCCGTCGAT	*Citrobacter farmeri* strain AUSMDU00008141: phage portal protein
spacer 9	CACCACATGAACCGCGACGCAGCGATTCACCG	
spacer 10	TTGCTAACTGGCGCGAAGAGCGCGCGTGGGCT	
spacer 11	ACAGTGAAATATATTGGGATAACATAAAGTCG	
spacer 12	GCGCACATATGAAAAAAGCCACGGGTTACGCG	
spacer 13	AAACGCGGGATTACGCGCCTGTTTGAAATCTGC	hypothetical protein
spacer 14	GCAATGCTCGTACGCCGTCTCACTCCGCGTGA	
spacer 15	CGCTACTATATGTGGTGTCGGGGCGGTGGTGG	
spacer 16	GCGGGCTGCGATTTCGCGATAGCTCGCCTTGC	
spacer 17	ATCCGGGCGGTATCTCAACGGGCAATGACGGC	
spacer 18	ACCCGAACGGCGAAGACGTTAACAAGGGCTGG	*Citrobacter amalonaticus* strain FDAARGOS_16S: phage major capsid protein, P2 family; *Salmonella enterica* serovar Seftenberg strain N17-509: phage major capsid protein, P2 family
spacer 19	TGCATACCGTGCCAGCGCCAGTGCGGCGCGGC	*Shigella sonnei* strain 2015C-3807 : plasmid unnamed1
spacer 20	GGACGTGTGCCGGAGATTTTGAACCGCAGTCA	
spacer 21	TGAATCTAAATGTTTTCCAATCTATTTTTTTG	
spacer 22	GCGTGCCAGCGCCTCTTCAAACCAGCAGCAGAA	
spacer 23	CAATTTTTTGAATTTGTTTTTTTCCAGAAATT	
spacer 24	GCAATATTATGGCGCTTCATTAGACTGAATAT	
spacer 25	AATCGTGTGTAAATTCGCGGCGGCTCCACTGG	
spacer 26	TTTTGTGCCCTGGCGTTTTGCTGGCTCTCGCG	
spacer 27	TTTGCCGCTGTCAGCATTGCTGGCGCGTAATA	
spacer 28	TCCGCATCGCTCATGCCGATAATCGGGCTCGG	
spacer 29	ATCAACGTTATCGATTACAACTGACAGGGAGC	
spacer 30	TATTTCTGTAATGCTCAAAGATATAGCCGGAT	
spacer 31	AAATATGGGGAGCGCCGTTAATGAAATCAAAA	
spacer 32	GAGGCAATTAGTAAAACTCCGGCAATTGCTGC	
spacer 33	CCGAACGGCTCGGCGCTCTCATACCCCCAAAT	
spacer 34	CCGGCGTTGAGCGCCAGATGACTGAGAAAGAG	
spacer 35	CTCACCGCTTTTGACGCCAGGGAAATCGGCGG	
spacer 36	GGTGTTCCGTTAATATCGATGGCGACGTAACT	
spacer 37	TTTTCTGAGGCTGTTTATGAGGATAAAAAATT	
spacer 38	GTCGCGTTTTTTCAAATTAACGCAATCGTAATC	

spacer 39	CGTGCCTTCGACTTTGCGGATCACGTCGTCTT	
spacer 40	GAGGTGGCAATACGCGTAGATCATTTGGTCGT	
spacer 41	CAAAATATTACGAGCTTCGTCAGGCCATGGAC	
spacer 42	ACGATGGCGATGCGTGAGAAAGGGGGTCGATA	
spacer 43	CCTAATTTTTATATAGCTGCGGATGATGTAAA	
spacer 44	TGCAACGGTAACTGGCTCACTGTTCCCCGCGG	
spacer 45	GATTTTAATAACTGTTGATCTACTCCTTGCAA	
spacer 46	GCGTTGATGTTCTTGCGGGTGTTGCAAATATT	
spacer 47	CGGAGCGTGTCTTGTGCTGCGTTACGGTAAAA	
spacer 48	AAAAGGGCCGCATTGACGGCCCTGTGTTATCG	
spacer 49	TTAGCTGTTAGCCAAACATGTCGACCTGTTTG	
spacer 50	GGGTGGCGGTGGTGCTGTAATTCACACCGGTA	
spacer 51	ACGGGGACGAATCACAGCAGCGTTATCGTGAT	
spacer 52	GTTTACCGCCCCGCAGAGGCGCTGGCAGATCCGT	
spacer 53	GGATGACCTGTCGCTAAAACTCGCCGCGTACAGT	
spacer 54	TCCGTTTGGTCCACCAAATGTTTGATGCTTCAGT	
spacer 55	TTCAGGGTGTGATTCCGCATACCAAGCAGTAAGA	
spacer 56	GGCGCACTGGATGCGATGATGGATATCACTTAGA	
spacer 57	CTGGCAGCACTGCGGGAAATATTGTTGCTGCT	
spacer 58	GCGGTAATTGCGTTATTTTGGCTGTTTTCCGC	
spacer 59	TCGTCGGGTCCAACAGGGGTCGGTTGTTCGCC	
spacer 60	GAGCTGATATTTTGATAACATCAACAGTTCAA	
spacer 61	ACATGAATGTCGGTTCAGACCGTGTTTTTACC	*Klebsiella pneumoniae* strain ST11: plasmid pKP12226; *Salmonella* phage SJ46; Enterobacteria phage P7
spacer 62	AGCGCGGGCTAATGTCTGCTCTGAATCATCAC	
spacer 63	CATGCTGCCGTCGTTTACGCCGTTCTTAATTT	
spacer 64	AACAGGCCGTATGAGGATCACTGGCACGGCGG	
spacer 65	TTTCTAGGAAACGACGTTATTTGCTCATCCCA	*Salmonella enterica* serovar Dublin strain HWS51: plasmid pATCC39184; *Salmonella enterica* serovar Pullorum strain ATCC9120: plasmid pCFSAN000725_01; *Salmonella enterica* serovar Gallinarum strain 287/91: plasmid pSG; *Salmonella enterica* serovar Pullorum: plasmid pSPUV; *Salmonella enterica* serovar Dublin strain 853: plasmid pSD_77; *Salmonella enterica* serovar Dublin strain CT_02021853: plasmid pCT02021853_74; *Salmonella enterica* serovar Dublin: plasmid pOU1115; *Salmonella enterica* strain OU7025: plasmid POU1113
spacer 66	ATCAGGTAATCGAGACACCTGTCAGCACTCTC	
spacer 67	GATATTTGGGCGTAATACCGCCCGGATAGTGT	
spacer 68	GTGAGCCATTACAGCGTGTACAGGCGGTCACG	
spacer 69	GGTAGTACGCGCCTCCGGACGTTTTTATGTCG	
spacer 70	TCTTCGCGGGTAATCAATGATGATTCAGTTTC	
spacer 71	AACATCGGAAACGGCTTCGCGGCGGCGGCGTC	
spacer 72	TTAGGCACAGAAAAAAAGGCTACTCAGCGAACT	
spacer 73	GGCATAGCCAGGCTGATCCGGCGACGGCCTTA	
spacer 74	TGGTCGAAATATAGACAGCATGTTCCGTACCA	
spacer 75	ACTACTGTCGGTAGCTGGGAGGATGAGGAGAT	
spacer 76	TGCCGCCAGGCCAGCGACACATCAGACAACTG	

spacer 77	GTCTGTGATGGCCTGCTCGTGAGTCCGCGGCG	
spacer 78	CCGGAGATCATCATATTCTGATACGTTCCGGT	
spacer 79	CGTTATTTACTGCTTTTTCATCCGCTCTGCTG	
spacer 80	ACTCCGATAGCCTGCTCAGAATTAAGGCGCAG	
spacer 81	CCGCCGTCGAGTGTAATACCCTGATCCATCGC	
spacer 82	GTGTGGTAATTGGTGGTCTGGCTGGTGGTTTA	
spacer 83	AGAATCTGGACACTGCATATACCGCAATCCGT	
spacer 84	CCAGCCGTTCAGTATTGCCGGTGTCAGCAAAA	*Citrobacter freundii* strain P10159: plasmid pP10159-4; Leclercia sp. LSNIH3: plasmid PLEC-7c0d; *Citrobacter freundii* complex sp. CFNIH9: plasmid pCFR-eb27; Enterobacteriaceae ENNIH1: plasmid pENT-1f0b; Leclercia sp. LSNIH1: plasmid pLEC-1cb1; *Salmonella enterica* serovar Enteritidis strain 81-1706: plasmid pSE81-1706; *Salmonella enterica* serovar Enteritidis strain 81-1705: plasmid pSE81-1705-1
spacer 85	GAAAAGCTACTTTTGTGTTCAACTGATGCATT	
spacer 86	TTGCAAACCGTGGCAAACGCAATTAACAAAAA	
spacer 87	ATTGTTATAATTATTTATTGAAATATCATTCC	
spacer 88	AATCTATTGTGAATTTGAAATGGTCCAGCACT	
spacer 89	GAGACCTGCCATGAGGTGGATTTATCAGTCGG	
spacer 90	TGCACGAAAAATGGCAGGCTTGCCAGCATCCT	
spacer 91	CCGCTTAACCGGACCTCATCACAAAACACGCG	
spacer 92	GCTATCGGGTGTGGATACCGCTTTCGAGGCGT	
spacer 93	GCAATTTGTTGTCCGCGATCCGGTACGCGCGT	
spacer 94	CCAAAGAAGAACAACGAGCCAACTGGTTTCAG	
spacer 95	GCGAAAAAAATACGGCTGGATGCGACAAAAAAA	
spacer 96	AATTTTCTTCATTGCTCAAACAGGCTGCATAAC	
spacer 97	GCGTCTCGAGCGCGGGACGATTCAAAACCAGC	
spacer 98	TTTATGTGGAGTATCAGTGTCTGGGTGGTAAG	
spacer 99	ACCTCGACAATGACACGGGCGCGAAAACAATC	
spacer 100	TCGGTGGCTGGATTGAAAAAGAAGATAATCTG	
spacer 101	GCAAATTACCGCCGGGCTAACGTTTGATCTCA	
spacer 102	ATGTCGAGCGCGTTGACAGCTATATTCGCCGT	
spacer 103	GCACTCAAAATAGTAAATTAATTTATGAATTG	
spacer 104	TTCAATGGTTGCCTCTTCATCATAATATAGAC	conserved hypothetical of bacteriophage origin
spacer 105	GGCCAGCAGGTCAAACGGATGTTCCACAATCA	
spacer 106	CAGGCCGGAGATCAGTTTAATACGCTGAT	
spacer 107	GGGGTAGAATTATTCTTCGTGAGCGATTTATC	
spacer 108	CGGCGTTCCGTGCGGCAATTGGAATCACACCA	
spacer 109	CGTTCTGAATCCGATATTCTTCAGCACCTTCA	
spacer 110	ATGTAGGGGCAATCGAACGATTCTCTGCCGAC	
spacer 111	GCGTCAATCAGCGCGTCTATCGCGTCACTTT	
spacer 112	CTATTGCTTTCGTACAGATTTTCAGTGGTGCT	
spacer 113	TCGAAGAAGAAAGGGAAATAATGCGAGGAACG	
spacer 114	CGTCACCACGCGCGCTACAGGTAATGCAGCTC	
spacer 115	GTTATTTTTTCACTAATTTCGGTTAGAGGGAT	
spacer 116	GCAATCACCAGCGGCGCGGCGTGGTTTGGT	
spacer 117	GATACCGCGATTCATGCGACGATAAAAAATAC	

spacer 118	GGGATGAAATTGAACTTCCAGGCGACCGACAA	
spacer 119	ATTGATAACTCGCGCTCATCCATTTTGAATTA	
spacer 120	ATACCCCAGCGTTTCGTTATATGACGGAAGAT	
spacer 121	GATTTTTCGCGAACTATCCCGGCGTAATGCGG	
spacer 122	ATCGCCCATGTAACAGAAACGGGGATTTTAATT	
spacer 123	GCTCTTCAACGGTTAGCTCAGGAGCTTCTTCTC	
spacer 124	GGTTTTACAAGCTTAAATGATTTTAAATTTTG	
spacer 125	AACTGGATTCTGCTCTAATTATTACATCATCG	
spacer 126	GGAAACCGCCCCGGCGGTGGTTAGCTGTTTGC	
spacer 127	TTTGCTACCCGCTCAAAACCGCCGATTCGCGT	
spacer 128	TCGGCCAGCGTCCAGGCTGGCACCCGATATTC	
spacer 129	CTATAATTTGTTTTTGCACCACTGTACCTCGT	
spacer 130	TCTTCGGGCGCGCGTTTTCGCAGACCAGCGAT	
spacer 131	ATGAAACGCCGGACTAATCCACATGGCGTAGG	
spacer 132	ATCCACATTTCCGCCGTTATGAATCGGTATTC	
spacer 133	GGTTTACCCAGTGGGGTGATTGAATATTTTGC	
spacer 134	TTACCTCGAATAATTAACGCAAAATATATTTG	
spacer 135	GCACGCGCGGGTTCCTCCAAACGCCCTTCAAAC	
spacer 136	ATCCTTTTAATTAATGGGGCCCCAAATGATTA	putative phage protein
spacer 137	TTCCTGTGCCCTGAGTTGCACCTGTTGTTCCG	putative phage protein
spacer 138	TCGTCCCTGGGAGCGGCTTCAAACCACTCTCT	
spacer 139	ATATCGCTATACGCACCAACCGTTTTCGGCGT	
spacer 140	GGGTTCCCGTTCACTGACGAAATTGAGTGCGA	
spacer 141	TCACATTTGCCCTGTTCCCGTTGGTCGCGCAG	
spacer 142	TTATTTTATTCAGCTCGCACAGGTATCGTCGC	
spacer 143	GCTCGTCTGCGTATTCGTAGCAGTCAACAATA	
spacer 144	TGGTTTGCTATCTGATGGAATTTGAACGGATG	
spacer 145	GACGTAACACCGGATCCGGCGCGTACTGCATT	hypothetical protein in predicted phage genome
spacer 146	GCCTGACATTGCAGACGTTACAAATTGAGAGGC	
spacer 147	TAATCACGTTTTAGCGCGCCCTCGTCCGGTTTC	
spacer 148	CAGGCAGAGAATGACTATTTTTCTACAAATCTC	
spacer 149	CCACCGTTTTCGCCCACCAGGGCGCACAACCC	
spacer 150	GAAAAAGAGAAGGTAGAGAAAGCGGAATCTGG	
spacer 151	CAGGTCTATCGGGCGATCAATAAAATCGGTCA	
spacer 152	GCGCACCGTTGCGTCGAAAAGGCGCTGGAGAT	putative phage capsid protein
spacer 153	GGGCAAATATAAATTCCAGCGTGCTTCATGAA	
spacer 154	CTGCGTAGCGACCTTTGCTCTCAATTTCGTTG	
spacer 155	GACCCACGCACACACTGCGCGCAGCCCTGATT	
spacer 156	GATTTTTTCCGCTTCTGACAAATGGTTTTTAT	
spacer 157	CCCGAACCCGGCAACATGCTCGCCGACCTGCG	
spacer 158	TTGATGAAGCGGCGCGTGTTATAGCTGATGGC	
spacer 159	GAGCCCTGCCAGAATGGGGCCTCTTTTGTAC	*Klebsiella pneumoniae* strain DA48896: plasmid p48896_1<br>*Shigella dysenteriae* strain CFSAN010956: plasmid unnamed<br>*Klebsiella pneumoniae* strain CRKP-1215: plasmid pCRKP-1215_2
spacer 160	ATTTACTGATTGTACCAGGTAGGAAAAGATC	
spacer 161	ATGATTGTCGATTTTTTCCGACAGGATTTT	

spacer 162	GTCACACTGCCGTGCATTATTACAGCCAGGC	
spacer 163	GACTCGGGCGGAAAATATTCACCGAACGCTGG	
spacer 164	GACTGCTCAGATTGGGAATTTGACCAGCGACC	
spacer 165	CGGTTTAAGTCAATCAAACCGTTGGGAAAACA	
spacer 166	CCGCTAATGCGCGTATCTCATGGAAGGGTGGA	
spacer 167	AATAACTCGCGTAAATGCTCTGCGGCGCTACG	
spacer 168	CCTCCTGTACCACGATCGCGCCCTCATACCCC	
spacer 169	GTCCCCGATCGGAATGGCGACATGCGATGCCT	
spacer 170	CAAAAAACGCCGCGTTTTTTGCATTTTATCGG	
spacer 171	AACAAGCTCGCCCGGCCCGTCCTCATCAAGGG	
spacer 172	CCACTGCACTGACTCTCGTGCGTACGCTGCGG	
spacer 173	GCTGGCAACTGAATGCCCGCCGTATTGCCTTT	
spacer 174	CAAAGGCCCGCCAATGCGGGCCGATTTATCCC	
spacer 175	CCGGACGAGAAATTCACGTTCCTGCATTTCAT	
spacer 176	ACGAAACGCAGTACGGTTTTGTCAGTTAACAC	
spacer 177	ACTCGACAGAACGGCCTCAGTAGTCTCGTCAGGCTCC	
spacer 178	GCTCTACGTGAAGAATATTTGCAACACCCGCAAGAAC	
spacer 179	ACACTCTGCCGGGTGAAACCACTCGCGGCAGATCTTGC	
spacer 180	GCCAGTGCCTGGTCATATGTGTAACACCTATC	
spacer 181	TTGCTGTATAGAGATTATCGGCGTGGGATTGT	
spacer 182	GTTTGCGGCTTGCAAACATTTTAATGCGTATT	
spacer 183	CCAGCCCGACACAAATAATGCAGCATTGCGCG	
spacer 184	TACCGCTGTTCATGCCGGAAATTGACCGGACC	
spacer 185	CTCATATTTCGCGTCAATCTCCACCAGG	
spacer 186	TGGTTTGAGCCGTTTAAAGTTGAACAGGCGAC	
spacer 187	GGACACGGATAACAGCTACCTGCAAAATTTAAT	
spacer 188	GGCAATCCGTTGGATTCATTGTTTTGTTGCTC	
spacer 189	ATCTTCTTGTTCAGGAACGTCAGTAGAGGTGT	
spacer 190	AGCATTAACCCCCACCAGCTCGACGTGTGTGG	
spacer 191	GCGGGCGTTAACGCGGTGATACTGTTTGACGG	
spacer 192	ACCGAGCGCCGCTGGGAGGCGTATCTCACGTT	
spacer 193	TGAATGATCGGAAAGACGCTGCAAAGGCAATG	
spacer 194	ATTAAAGGATTATTTTGATGAGTCTGAAAAAT	
spacer 195	CTCAACCGCGCTTCCCGGCCTCACCACTGC	
spacer 196	TGAGCGTCGGCGGCTCGCTGGATTTGCGCGG	
spacer 197	CTGTCCCAGGCCGAGGCTGTATTTCAATCCTG	
spacer 198	ACCATTACTTCGGTAAAAGCGTGATCAAGAAT	
spacer 199	TCGATCAGGCGGTTTTGTTCTTCCAGCGACGC	
spacer 200	AAAATTCATATTGATAAACACCGCGTTTGTAT	
spacer 201	TTTACTACAATAGGTGGACACGCTACGGCGCA	
spacer 202	CCGTTCATATTCGTTTCCTCGTGGCGCGATCT	
spacer 203	GGACCAAATACCCGTCCCTCAATCCACGCCGT	
spacer 204	TGACAAATAGAAATCATATGATACTATAGCCA	
spacer 205	ATCATCTCCGCTGAATAGCGTAAATTATCAGG	
spacer 206	ATTAAATCGTCAGAAAATAGCGGTAATCAAGT	
spacer 207	GGCGGCGGTTTATTGTCAATGTGCAGCCCCAG	

spacer 208	ATATCGCGCGGGTCGCTAATTTTTTTACCCCG	
spacer 209	GGGAATTCAGCAAGAATACCGCGTTTATAACT	
spacer 210	CAGTACCAATCCAGCATTTATCCCAGACAATA	
spacer 211	TACGGCTCAATTGATCGCCCCGCCTCAAATTC	
spacer 212	GGCCTATGCGTTTTTCTCTCTGTTAATGCGCCCC	
spacer 213	GAACGAAATATCACGGCGTTCTGCGGCCTCGG	
spacer 214	ATTATTGACCATTTCGCCGGTAAGATTAGCCG	
spacer 215	TCACGCGCTGTTACCCAGTCCCTTTTTTTCA	
spacer 216	TACAGGAAAGAATCCGCCAACGGCGACAGGG	
spacer 217	GAATATCCACCCGTCCTGTATTCCGCCAATT	
spacer 218	TACCAAACCGGAATCTTTCCATATAACGGCG	
spacer 219	ACCCCGAAATTAATGGCGACGGGGCGTTTACG	
spacer 220	ATCAAACTGCTGCAACGGCCAGCGCAGCCACG	
spacer 221	TGCCTCGGCGGCGGCTCTGCGCAACCGCGAAA	
spacer 222	TCGTTTTGTTAGCCAGCCGTCGCGCACCAGTT	
spacer 223	GTATTGAATCGTGTGTAAATTCGCGGCGGCCC	
spacer 224	TGCGTGATCCCCTCGCTAACGTACATATCGAT	
spacer 225	GCAAAACCTGTCGATCTTGATACTGGTCGCTC	
spacer 226	TTGCTGCAATTTTGTCTTTTAATTTATCCTAG	
spacer 227	CGGACACCGTACACAGATTTATATCAATCATT	
spacer 228	TCTTTCGCAGCATCTGGAACTAAATTAATTGC	
spacer 229	ACAGCGCAAACCGGAGATCAAACCGGCGGAGC	
spacer 230	GTCGATAATATTTTGCTGTTGTTTTGTTACGT	
spacer 231	CAAAACCAAACTTCTCCATAAATTCCATAGCCG	
spacer 232	CGTCGGGCCGGGTAAGTGAACCTGTCGGTTTTC	
spacer 233	GTTAAACGCCTGCTCGACCCTGTCATGCGGCG	
spacer 234	ACTTATCGCACCGTGTTAAACCGGCAGAAAAACA	
spacer 235	ACCTAAACAGAGGTGTAATACCGGTGAAAATGCC	
spacer 236	CTCAAGATAGAGCCTCCGGGTCGAGCCGCGAT	
spacer 237	AAAATTCTGTGTTTCGACCATTACTTCGGTAA	
spacer 238	CGGCTTGTTTAATTGCGTGGAACGTCTCAATT	
spacer 239	GCAAATTAAGCTGGCTGGCAATCTCTTTCGGG	
spacer 240	TTTCAGCAGTTCAGCGTAACACCGACGGTCAC	
spacer 241	GCTGTTTGTAAATTTAGAATTGCTGACTGAGC	
spacer 242	TCGTCGTTTATTATTGATGCTGATGTTTTTGCC	
spacer 243	TAATCAAAAGCGGCCAGAACTGCCATTTC	
spacer 244	GGGATTTGCATTAACGCGGCGTGAACATTATTT	
spacer 245	TTATATTTATTCGACGCTGACAGGCTCACAGG	
spacer 246	GCGAGGGGCAGCCGTTCGCGCTGCATGTTGAT	
spacer 247	AAAAAACAGTGGTACTACCGCCCCGCCGAACA	
spacer 248	TTAACGTCTGGATCCTGTGTGCCATGGACCGT	
spacer 249	GATCGCCGGGCTCGACTACCCGCCGCGAAAAT	
spacer 250	GCTGTTAGACTACATATCCTCGCGCGTGCGCC	
spacer 251	GTGGTGATAATCCGATTTGAATAGGACTATCC	
spacer 252	CTGTGCTCCTGTTTTGTGCCGTAGTTACCAAT	
spacer 253	TTTGAGAATTGTCGATGAATGATCCTAAATGG	

spacer 254	TCAGCGGCGGTGATCCCTGAGGTACCAAACAT	*Citrobacter freundii* strain P10159: plasmid pP10159-4; Leclercia sp. LSNIH3: plasmid pLEC-7c0d; Enterobacteriaceae ENNIH1: plasmid pENT-1f0b; *Klebsiella oxytoca* strain CAV1374: plasmid pKPC_CAV1374; *Salmonella enterica* strain 8025: plasmid p8025
spacer 255	TTTTCCAGATAACTAACGCTTTCGCGTGCATG	
spacer 256	TCGATCATGCGCCTGACGTCGCTCGCAGACTC	
spacer 257	GGCTATTGGGTTATTAAAGACCTGAATGAAAC	
spacer 258	CGGCGACGACTGAAAAGGCGAGGCAATTAATT	
spacer 259	CCGGATTAATAACTGGCAACATGCCGCGCAAT	
spacer 260	AGTTCGGTAAGTATCGTGGATGTAGCTGTAAT	
spacer 261	CAAAACCAATAGGTGCTTTCCACATTGCAGAA	
spacer 262	GTTCTCCAATCAGCAGTGCCCATCTGATATGCA	*Salmonella* phage UPF_BP1
spacer 263	GTCAGGGGAATTGCGATATTCAGGCAGCGTAAC	
spacer 264	GCCCGCCGCCGCTGTCGTTCGGTAGTGTCCGG	
spacer 265	GGGGATAACGGTTATCCACTGGCCGCCGATCT	
spacer 266	TGTAATAGCCTGATCTCTGATCTCCCTCGCCT	
spacer 267	TCCTCATGTAATTCCTGTGCCAACTCAATAAG	
spacer 268	AGATATCTGTTCCGGCTTCCAGCGTTTTGTTG	
spacer 269	CGGCAACATAACGAACAAAATCAACGTCAACCT	
spacer 270	CTTGCTGAAAAAGAAGGCTCCGGCGTTATCAGT	
spacer 271	TATGCCTCTTTTAAACGCGCCGCCTTTTTGCC	
spacer 272	TGAATGATCCCAAATTTGGGTTACAGAACCAGT	
spacer 273	AATTTTTTTACCCCGTTTAGCGCCGGGGAAAA	
spacer 274	TCAATTTGTTCTCGTATCCAGGTGTGATCTAA	
spacer 275	CCCGGAATGCATTCTGAAGGTTTGCTGTATAT	
spacer 276	ATCTCCTGCACCAGCCCCCATCAAATCAGCGT	
spacer 277	TTCGCGGTATACGCATTATAAGGCGCAACAGG	
spacer 278	CTCAGGAATTGATATTTTTGCCCCCTGCGAAC	
spacer 279	TCGCGGAATACACCGACGAGGCGGGCAAAAAA	
spacer 280	AGCCGATCCTCGCTTTCTGTTTCCAGAATTAC	
spacer 281	AGTCAGGCCGTAAAGCGTGATTGTTGGGACCT	
spacer 282	GGCAATCACATTGCCCTGGTTGACGAGGGGCG	
spacer 283	ATAACCCATTATTCCAACAACAAACGCTATTT	
spacer 284	ATTCAGAGCAGACATTAGCCCGCGCTGTCTGG	
spacer 285	GGCATTGACGCTTTAAACGACGACGGACGCCACGT	
spacer 286	TGTGACTTAAAGATCGCATCACAGTACTGGAGGG	
spacer 287	GCGCTCACATTTAGCGCCAGAGCCAGGCGGCG	
spacer 288	AATTCGGAATCACATTAAAAGGGAACCCGATA	
spacer 289	TTACCGAGCGTCACGAAACAGACAACACCAGG	
spacer 290	GCCCAGAATCTCTTTTAGCGCAACGATATTCC	
spacer 291	GTAACGTCTGCCTTTTCCTCGTCCGCCTTAAT	
spacer 292	CCGCCGCAACAATCGCGCCCGCAGTGCTGCCC	
spacer 293	GTTGTATCCGGCCTGTCGTTCACTCCCGCAGT	
spacer 294	TTCAGTGAGCCATAAAACCGCAGCTTATTTGC	
spacer 295	CAAAAAAATAGATTGGAAAACATTTAGATTCA	

spacer 296	TTGCATCATTTTTGTACACGTCGAGCTTCCCC	
spacer 297	CGCGTTGAAAGCGCGAGCGCGTTAGCTGACGA	
spacer 298	CTCACGGGCTATTTGGTTAATCGAAAATTA	
spacer 299	ACTGCGGGAGTGAACGACAGACCAGACACCAC	
spacer 300	GTAAACACAAGAAAAGGGCGGCACGGAGCCGCC	
spacer 301	GGCATATTTCATGCAGCGTTCTGAATGAGTAAG	
spacer 302	GGCGTGGCGCGGGGCGTATTCCCCGGCGAGTAA	
spacer 303	CGGCCGGTGGATAATGCGATTTTGTTACGCGG	
spacer 304	CCTCGCCAGATATCACCGAATCCCACCCGGTA	
spacer 305	GAGCGTAGAGATTCTCGAAACCGATCCGGACG	
spacer 306	AATAAATAATAATCCAGTAGCCCCATGTATTA	
spacer 307	GTAATAAAAAATATTCTATTTCTGCTGAATCA	
spacer 308	CTTGCGAGCAAAAGATCCAGGCCGAGAAAGAC	
spacer 309	CGATAACCGCGTCAGTATTGGACAGGATATCA	
spacer 310	GTGCTGCGACCTCAGCCTGACTTGCCATTAAA	
spacer 311	GCGCGAATTTGTGCGCATGGGGCGCATTTTTGGC	
spacer 312	AACTATTCTCTGATCCTTAAGCGATTGAAGAG	
spacer 313	GGCTTTAAAATTGTGTTCCCGTCTGGCGCTGGG	
spacer 314	GCATCCATGCCGACGCCTTTACGTGTGCGGGGC	
spacer 315	CTATAGCGCCACGTTCCGAGCGCTGCGAGCTGC	
spacer 316	CTAATGAGTCAGTTTGAACAGATCCCCGACAAC	
spacer 317	GAGTCTATCAGCGACACTACCGGCAATAGCGACTC	
spacer 318	CGAAATTGCCGACAGTAATAAAATGGAATTTT	
spacer 319	TGCAAAACAAAACTGTATTGATCGCGTTTTGT	
spacer 320	GTTTCGTAGCTCATTTTTGTAGCATTTTTCTC	
spacer 321	TATCAAGAAATAGTCGAAGTCTTCATGACTTC	
spacer 322	CCGGGATATTGCCCACCAGCTCCGCTACTGTT	
spacer 323	GGAGAAAAGTACCTGGTAAGCCGTGGGATAAC	
spacer 324	ATGACGACCCATGACAGATGGCGTTTAACGTA	
spacer 325	ATCATATTTATGAAATTCGCTATTTGCTGAAT	
spacer 326	CTGTGAGTTATTCAAATGGGCGTCCAGTGATG	
spacer 327	ATAACTAAGAGCACCACAATGATGATAAATTT	
spacer 328	CGCGTGTTTTGCGATGAAGTTCGGTTAAGTGG	
spacer 329	GCAAGCCGCTCATTTGACGGTCACGCCAGTTAC	
spacer 330	CTAGTTAACTTTTAGACAGAATATCCGTGTACC	
spacer 331	GAAGTTGCGCGCACTAAGAAAAACAGGTTCGA	
spacer 332	CTGAAATCAATCGCCGGTCGTCGCCACGTTAC	
spacer 333	TCATCGAAAACCAGCGGATCGAATGGGACGCC	
spacer 334	AGCCGGCGCGTAAAACTCATACGGCGACAAAT	
spacer 335	CTCGCGGGCGTGGAATTTAACGAACGGTTACC	
spacer 336	TGGGTGAGCCAGCGCCGCAGGAAGACACGC	
spacer 337	CGTTCCCAACGCTGAATATTGCTGGCATAGCC	
spacer 338	TGGCGATATCACCTGATGCCTGCAATCC	
spacer 339	GGGTATCGCACTGCGGCAGATGTTCCGGGGCC	
spacer 340	CATGAATATGGACGATGAAAAAATAAGAGAGG	
spacer 341	TTAAAACATTTTCCGAGCTTTGCGATTTATAT	

spacer 342	TAAAAATCATCATGGAAAATCAACCCGGCGAC	
spacer 343	TAACGCCCACGCCTCAACGCGGCACATAAAAT	
spacer 344	GAGCTGGCGATAGCCCTGAATGAACTGGCAGA	
spacer 345	GCGAATACATGGAGTTAAACAGGATGTGGCTGC	
spacer 346	ATCAGTGATATCCAACATTGCGGAGCTAAACG	
spacer 347	TGCCACTGGTTTCATGCAGGCCGCGCAAAAAT	
spacer 348	CATCGACTTATGCCCCATCAGGCTCTGCAATAC	*Klebsiella oxytoca* strain KONIH2: plasmid pKOR-e6bf
spacer 349	CAATTCATATACTGATAAATCATCAAAACAAA	
spacer 350	CCCCTGCGCTTGATGAACGACAACAAGAATGG	
spacer 351	GCAGTACCATTACATCAAAGAGTGCAGAATCC	
spacer 352	GGTGCTGTCTTTACCGTACATTGCGCGAATCT	
spacer 353	ATATCCTTATTGGAATACATGAGCTGCGCCCA	
spacer 354	CGCGACACGCTGGCAGAGCAGCTATCCCACCA	
spacer 355	TTAATCAGGTCGTCGTACTCAGCGCCGCTGGC	
spacer 356	AGCACGGCAGGCCATATGAAATACCTGTTGCT	*Escherichia coli* O157 typing phage 10; Enterobacteria phage HK629/phiV10
spacer 357	GCACCCATCAAGGAATAACGCGTTTATTTTCC	
spacer 358	TAATATCCCTGGCGATAATCAACCGGCTTACT	
spacer 359	GAGAGCGAGGGGAGATTCCGGCAGGGCGTGCA	
spacer 360	TTTAGCGGCCCCGCCTTTCAGCCCTGGTGCCG	
spacer 361	TCAATAATCTGATTACCGAACCCCAGCCCCAA	
