# Supplementary material for: Genetic Diversity of Non-O157 Shiga Toxin-Producing Escherichia coli Recovered From Patients in Michigan and Connecticut
Source: Front Microbiol. 2020 Mar 31;11:529. doi: 10.3389/fmicb.2020.00529 (PMC7145412; doi:10.3389/fmicb.2020.00529)
Supplement: Supplementary file 3 [file Data_Sheet_3.PDF]

Table S3: CRISPR spacer profiles based on the concatenated sequences for CRISPR1 and CRISPR2 among 149 non-O157 Shiga toxin-producing *Escherichia coli* strains. The first four columns represent the accession number, serogroup, H-antigen type, and sequence type (ST), respectively. Strains belonging to Cluster 1 are represented with orange shading in the ST box and Cluster 2 strains have blue shading. Strains with white shading in the ST box grouped together with strains outside of Clusters 1 and 2 in the MLST analysis. The spacers are indicated in the colored boxes along with each spacer number that refers to a specific DNA sequence (Table S2); identical spacers are indicated by the same color and number. The count refers to the total number of spacers detected in both CRISPR loci and profile represents the profile number for each unique spacer combination.

Strain	O type	H type	ST	Count	Profile	CRISPR spacer profile																																									
TW10118	O145	H28	78	10	1	56	89	90	155	156	157	158	159	160		162																															
TW14002	O145	H28	78	11	2	56	89	90	155	156	157	158	159	160	161	162																															
TW14920	O145	H28	78	11	2	56	89	90	155	156	157	158	159	160	161	162																															
TW14943	O145	H28	78	11	2	56	89	90	155	156	157	158	159	160	161	162																															
TW14952	O145	H28	78	11	2	56	89	90	155	156	157	158	159	160	161	162																															
TW14998	O145	H28	80	6	3		89	90					159	160	161	162																															
TW07612	O5	H9	175	11	4	211	210	209	280	292	293	279	278	55	56	71																															
TW14911	O5	H9	175	9	5	211	210	209	280			279	278	55	56	71																															
TW14931	O5	H9	175	10	6	210	211		280	292	293	279		55	56	71	294																														
TW11539	O49	H16	352	9	7						293			55	56		294	235	303	304	177	73																									
TW14917	O174	H21	89	34	8	145	144	231	142	141	140	139		137	136	135		133	132			56			130	129	128	127	126	125	124	330	270	269	97	148	147	146	94	93	264	287	288	289	290			
TW14933	O146	H21	845	33	9	145	144	143	142	141	140	139	138	137	136	135	134	133	132	131	315	187	262	263	130	129	128	127	126	125	124	330	270	269	97	148	147	146									
TW14934	O146	H21	845	33	9	145	144	143	142	141	140	139	138	137	136	135	134	133	132	131	315	187	262	263	130	129	128	127	126	125	124	330	270	269	97	148	147	146									
TW14937	O146	H21	845	33	9	145	144	143	142	141	140	139	138	137	136	135	134	133	132	131	315	187	262	263	130	129	128	127	126	125	124	330	270	269	97	148	147	146									
TW09182	O103	H2	119	13	10			231	317					315	39	147	146	51	234	272	286			40	72	42																					
TW14932	O103	H2	119	15	11			231	317					315	39	147	146	51	234	272	286		41	40	72	42					3																
TW14983	O103	H2	119	15	12			231	317					315	39	147	146	51	234	272	286			42	72	40			314		3																
TW11542	O103	H2	119	13	13			231	317					315	39	147	146	51	234	272			41	40	72	42																					
TW14951	O103	H2	119	13	14			231	317					315	39	147	146	51			286		41	40	72	42					3																
TW15003	O103	H2	119	12	15			231	317						39	147	146	51	234	272	286				72	40					3																
TW11537	O103	H2	119	19	16			231	317					315	39	147	146	51	234	272	286		41	40	72	42	316	314	313	311	3																
TW14914	O103	H2	119	19	16			231	317					315	39	147	146	51	234	272	286		41	40	72	42	316	314	313	311	3																
TW14915	O103	H2	119	19	16			231	317					315	39	147	146	51	234	272	286		41	40	72	42	316	314	313	311	3																
TW14926	O103	H2	119	19	16			231	317					315	39	147	146	51	234	272	286		41	40	72	42	316	314	313	311	3																
TW14967	O103	H2	119	19	16			231	317					315	39	147	146	51	234	272	286		41	40	72	42	316	314	313	311	3																
TW14987	O103	H2	119	19	16			231	317					315	39	147	146	51	234	272	286		41	40	72	42	316	314	313	311	3																
TW14989	O103	H2	851	19	16			231	317					315	39	147	146	51	234	272	286		41	40	72	42	316	314	313	311	3																
TW14992	O103	H2	119	19	16			231	317					315	39	147	146	51	234	272	286		41	40	72	42	316	314	313	311	3																
TW14910	O103	H2	119	20	17			231	317					315	39	147	146	51	234	272	286	56	41	40	72	42	316	314	313	311	3																
TW14907	O103	H2	119	18	18			231	317					315	39	147	146	51	234	272	286			40	72	42	316	314	313	311	3																
TW15002	O153	H2	119	18	18			231	317					315	39	147	146	51	234	272	286			40	72	42	316	314	313	311	3																
TW14966	O103	H2	119	17	19			231	317					315	39	147	146		234	272	286			40	72	42	316	314	313	311	3																
TW14994	O103	H2	119	17	20			231	317					315	39	147	146	51	234		286		41	40	72	42	316	314	313	311																	
TW14941	O103	H2	119	18	21			231	317					315	39	147	146	51	234	272	286	56		40		42	316	314	313	311	3																
TW14902	O103	H2	119	18	22			231	317					315	39	147	146	51		272	286	56		40	72	42	316	314	313	311	3																
TW09183	O45	H2	119	11	23			231	317					315	39	147	146	51	234	272	286	56																									
TW09370	O45	H2	119	11	23			231	317					315	39	147	146	51	234	272	286	56																									
TW10117	O45	H2	119	11	23			231	317					315	39	147	146	51	234	272	286	56																									
TW11541	O45	H2	119	11	23			231	317					315	39	147	146	51	234	272	286	56																									
TW11543	O45	H2	119	11	23			231	317					315	39	147	146	51	234	272	286	56																									
TW11544	O45	H2	119	11	23			231	317					315	39	147	146	51	234	272	286	56																									
TW11564	O45	H2	119	11	23			231	317					315	39	147	146	51	234	272	286	56																									
TW14958	O45	H2	119	11	23			231	317					315	39	147	146	51	234	272	286	56																									
TW09373	O45	H2	119	10	24			231	317					315	39	147	146	51	234	272	286																										
TW10121	O45	H2	119	10	24			231	317					315	39	147	146	51	234	272	286																										
TW14003	O45	H2	119	10	24			231	317					315	39	147	146	51	234	272	286																										
TW14329	O45	H2	119	10	24			231	317					315	39	147	146	51	234	272	286																										
TW14901	O45	H2	119	10	24			231	317					315	39	147	146	51	234	272	286																										
TW14916	O45	H2	119	10	24			231	317					315	39	147	146	51	234	272	286																										
TW14918	O45	H2	119	10	24			231	317					315	39	147	146	51	234	272	286																										
TW14922	O45	H2	119	10	24			231	317					315	39	147	146	51	234	272	286																										
TW14925	O45	H2	119	10	24			231	317					315	39	147	146	51	234	272	286																										
TW14927	O45	H2	119	10	24			231	317					315	39	147	146	51	234	272	286																										
TW14928	O45	H2	119	10	24			231	317					315	39	147	146	51	234	272	286																										
TW14942	O45	H2	119	10	24			231	317					315	39	147	146	51	234	272	286																										
TW14946	O45	H2	119	10	24			231	317					315	39	147	146	51	234	272	286																										
TW14963	O45	H2	119	10	24			231	317					315	39	147	146	51	234	272	286																										
TW14964	O45	H2	119	10	24			231	317					315	39	147	146	51	234	272	286																										
TW14975	O45	H2	119	10	24			231	317					315	39	147	146	51	234	272	286																										
TW14988	O45	H2	119	10	24			231	317					315	39	147	146	51	234	272	286																										
TW14991	O45	H2	119	10	24			231	317					315	39	147	146	51	234	272	286																										
TW14995	O45	H2	119	10	24			231	317					315	39	147	146	51	234	272	286																										
TW15004	O45	H2	119	10	24			231	317					315	39	147	146	51	234	272	286																										
TW15010	O45	H2	119	10	24			231	317					315	39	147	146	51	234	272	286																										
TW14669	O45	H2	119	9	25			231	317					315	39	147	146	51	234	272																											
TW15005	O103	H2	119	11	26			231	317					315	39	147	146	51	234	272	286										3																
TW14979	O103	H2	119	12	27			231	317					315	39	147	146	51	234	272	286	56									3																
TW14923	O45	H2	119	8	28			231	317					315	39	147	146	51				56																									
TW14623	O45	H2	119	7	29			231	317					315					234	272	286	56																									
TW14912	O8	H14	286	27	30		213	231	317	66				315	39	147	146	51	234			56	69	70								314	311	214	281	282			147	212	316	313	3	1	176	283	284	
TW14001	O103	H2	526	11	31			231	317					315		147	146		234							42	316	313	314	311																	
TW14929	O103	H2	119	11	32			231	317					315									41	40	72	42	316	313	314	311																	
TW14330	O103	H2	772	14	33				317					315									41	40	72	42	316	313	314	311		180	181	182	183												
TW15017	O103	H2	119	9	34			231	317					315			146					56	41	40	72	42																					
TW14921	O88	H25	150	12	35		213	231							39	147	146					56		70											40	72		248	311									291
TW09177	O76	H19	91	21	36	56	55	54	53	52	146	147	51	39	3	272	286	50	49	48	47	236	60	59	58	57																					
TW14970	O76	H19	91	15	37	47	48	49	50	272	3	39	51	147	146	52	53	54	55	56																											
TW07613	O113	H21	161	43	38	100	99	98	14	101	102	103	104	105	13	12	11	10	9	8	7	273	6	5	4	274	217	146	147	51	39	234	3	2	1	176	272	286	175	174	173	172	171	188	167	168	169	170
TW07619	O110	H28	162	40	39	100	99	98	14	101	102	103	104	105	13	12	11	10	9	8	7	273	6	5	4			146	147	51	39	234	3	2	1	176	272	286	175	174	173	172	171		167	168	169	170
TW14962	O118	H16	106	6	40			231				178			69	70		73				25																									
TW14609	O151	H8	310	21	41	232		231	317			178	177	56			71	73	74	75		25				40	72	42	248	311	212	322	315	246	247												
TW11540	O111	H8	106	10	42	232	213	231		66	177	178	179								67			166	68																						
TW14903	O111	H8	106	10	42	232	213	231		66	177	178	179								67			166	68																						
TW14968	O111	H8	106	10	42	232	213	231		66	177	178	179								67			166	68																						
TW14996	O111	H8	106	10	42	232	213	231		66	177	178	179								67			166	68																						
TW14997	O111	H8	106	10	42	232	213	231		66	177	178	179								67			166	68																						
TW15006	O111	H8	852	10	42	232	213	231		66	177	178	179								67			166	68																						
TW14913	O111	H8	106	11	43	232	213	231		66	177	178	179	56							67			166	68																						
TW14956	O111	H8	106	11	43	232	213	231		66	177	178	179	56							67			166	68																						
TW14957	O111	H8	106	11	43	232	213	231		66	177	178	179	56							67			166	68																						
TW14972	O111	H8	106	11	43	232	213	231		66	177	178	179	56							67			166	68																						
TW14973	O111	H8	106	11	43	232	213	231		66	177	178	179	56							67			166	68																						
TW14999	O111	H8	106	11	43	232	213	231		66	177	178	179	56							67			166	68																						
TW15015	O111	H8	106	11	43	232	213	231		66	177	178	179	56							67			166	68																						
TW14993	O111	H8	106	11	43	232	213	231		66	177	178	179	56							67			166	68																						
TW14936	O111	H8	106	11	43	232	213	231		66	177	178	179	56							67			166	68																						
TW14944	O111	H8	106	11	43	232	213	231		66	177	178	179	56							67			166	68																						
TW15011	O111	H8	106	11	44		213	231		66	177	178	179	56							67			166	68	166																					
TW15012	O111	H8	106	10	45		213	231		66	177	178	179	56							67			166	68																						
TW14361	O111	H8	106	10	46	232	213	231		66	177	178	179	56							67			166																							
TW14955	O111	H8	106	9	47	232	213				177	178	179	56							67			166	68																						
TW15014	O111	H8	106	8	48		213	231			177	178	179	56							67				68																						
TW09372	O111	H8	106	7	49	232	213	231		66	177	178	179																																		
TW14960	O111	H11	106	10	50	232	213	231		66	177	178	179	56												232	231																				
TW14947	O111	H8	106	7	51	232	213	231		66			179								67				68																						
TW10130	O26	H11	106	16	52	232	213	231	317	66	177	178	179		69	70	71	73	74	75	67	25																									
TW11365	O26	H11	106	16	52	232	213	231	317	66	177	178	179		69	70	71	73	74	75	67	25																									
TW14670	O26	H11	106	16	52	232	213	231	317	66	177	178	179		69	70	71	73	74	75	67	25																									
TW14935	O26	H11	106	16	52	232	213	231	317	66	177	178	179		69	70	71	73	74	75	67	25																									
TW14974	O26	H11	106	16	52	232	213	231	317	66	177	178	179		69	70	71	73	74	75	67	25																									

Strain	O type	H type	ST	Count	Profile	CRISPR spacer profile																																																			
TW14984	O26	H11	106	16	52	232	213	231	317	66	177	178	179		69	70	71	73	74	75	67	25																																								
TW11538	O26	H11	106	17	53	232	213	231	317	66	177	178	179	56	69	70	71	73	74	75	67	25																																								
TW09184	O26	H11	106	17	53	232	213	231	317	66	177	178	179	56	69	70	71	73	74	75	67	25																																								
TW14949	O26	H11	106	17	53	232	213	231	317	66	177	178	179	56	69	70	71	73	74	75	67	25																																								
TW14961	O26	H11	106	17	53	232	213	231	317	66	177	178	179	56	69	70	71	73	74	75	67	25																																								
TW14977	O26	H11	106	17	53	232	213	231	317	66	177	178	179	56	69	70	71	73	74	75	67	25																																								
TW14985	O26	H11	106	17	53	232	213	231	317	66	177	178	179	56	69	70	71	73	74	75	67	25																																								
TW10239	O69	H11	104	17	54	232	213	231	317	66	177	178	179		69	70	71	73	74	75	67	25	41																																							
TW14939	O26	H11	106	17	54	232	213	231	317	66	177	178	179		69	70	71	73	74	75	67	25	41																																							
TW14948	O26	H11	106	17	54	232	213	231	317	66	177	178	179		69	70	71	73	74	75	67	25	41																																							
TW15008	O103	H2	119	17	54	232	213	231	317	66	177	178	179		69	70	71	73	74	75	67	25	41																																							
TW14953	O118	H16	106	18	55	232	213	231	317	66	177	178	179	56	69	70	71	73	74	75	67	25	41																																							
TW15009	O26	H11	104	18	55	232	213	231	317	66	177	178	179	56	69	70	71	73	74	75	67	25	41																																							
TW15013	O26	H11	104	18	55	232	213	231	317	66	177	178	179	56	69	70	71	73	74	75	67	25	41																																							
TW14924	O26	H11	844	16	56	232	213	231	317	66	177		179	56	69	70	71	73	74	75	67	25																																								
TW14981	O26	H11	106	14	57	232	213	231	317	66	177	178	179				71	73	74	75	67	25																																								
TW15016	O26	H11	106	15	58	232	213	231	317	66	177	178	179				71	73	74	75	67	25	41																																							
TW15000	O91	H14	815	14	59	232		231	317	66	177	178	179	56			71	73	74	75	67	25																																								
TW15001	O26	H11	106	13	60	232		231	317	66	177	178	179				71	73	74	75	67	25																																								
TW14909	O26	H11	106	12	61	232	213	231			177	178	179				71	73	74	75	67	25																																								
TW15007	O26	H11	106	14	62	232	213	231	317	66	177	178		56	69	70	71	73				25	41																																							
TW14919	O103	H2	106	11	63	232	213	231	317	66			179		69	70	71			75			41																																							
TW10122	O26	H11	338	7	64	232	213	70	69	66	317	231																																																
TW14930	O26	H11	104	7	65	232	213	231			177				69	70	71																																												
TW14938	O111	H8	865	18	66	232	213	346	347	348	349	350	351	352	353	354	355	356	357	358	359	360	361																																							
TW14969	O84	H2	158	10	67	240	275	100			45			56			146	147	43	44	46																																								
TW14954	O103	H25	159	17	68	240	163	164	102	14	98	165	95	123	94	240	99	147	43	44	46	101																																								
TW14945	O121	H19	182	6	69	240	275	218	217	216	215																																																	
TW14959	O121	H19	182	9	70	240	275	218	217	216	215	297	296								46																																								
TW14965	O121	H19	182	10	71	240	275	218	217	216	215	297	296	56							46																																								
TW10124	O91	H14	339	16	72	240	275	231	93	94	123	95			270	40	276	277	92	96	122	97	271																																							
TW14905	O91	H14	815	21	73	240	275	231	93	94	123	95	91	56	270	40	276	277	92	96	122	97	271	269	40	195																																		
TW14978	O126	H27	848	20	74	324	325	326	327	328	329	249	250	251	252	253	254	255	256	40	257	258	259	260	261																																			
TW14971	O33	H4	4	17	75	61	62	63	64	65	219	220	221	222	223	224	225	226	227	228	229	230																																								
TW07611	O22	H8	145	16	76	61	76	77	37	36	78	79	80	81	82	83	84	85	86	87	88																																								
TW14908	O163	H19	149	28	77	61	196	197	198	201	200		199	81	82	83	84	85	86	87	88	29	110	28	113		26	202	203	204	205	206	207	85	84																						
TW10123	O187	H52	274	25	78									112	237	198	107	108	109	111	33	29	110		113													117			115				120	244	116	114	118	119	77	300		302	106	30	121
TW11536	O145	H28	274	49	79	56	238	78	31	201	200	239	199	112	237	198	107	108	109	111	33	29	110	28	113	27	26	23	22	21	20	19	298	18	17	16	242	117	129	9	115	241	320	243	120	244	116	114	118	119	77	300	301	302			
TW14950	O13	H21	846	8	80	149	150	151	152	153	154	285	26																																												
